# Long noncoding RNA LINC00930 promotes PFKFB3-mediated tumor glycolysis and cell proliferation in nasopharyngeal carcinoma

**DOI:** 10.1186/s13046-022-02282-9

**Published:** 2022-02-24

**Authors:** Baoyu He, Hongli Pan, Fengque Zheng, Saiqiong Chen, Qingli Bie, Jinghe Cao, Rou Zhao, Jing Liang, Li Wei, Jianchao Zeng, Hui Li, Xing Cui, Yixuan Ding, Wei Chao, Tiantian Xiang, Yuhe Cheng, Gui Qiu, Shishun Huang, Libo Tang, Jiansheng Chang, Delan Luo, Jie Yang, Bin Zhang

**Affiliations:** 1 Department of Laboratory Medicine, Affiliated Hospital of Jining Medical University, Jining Medical University, Jining, Shandong China; 2 Medical Science Laboratory, the Fourth Affiliated Hospital of Guangxi Medical University, Liuzhou, Guangxi China; 3 Department of Reproductive Center, Affiliated Hospital of Jining Medical University, Jining Medical University, Jining, Shandong China; 4 Department of Obstetrics and Gynecology, The Fourth Affiliated Hospital of Guangxi Medical University, Liuzhou, Guangxi China; 5 Department of Oncology, the First Affiliated Hospital of Guangxi Medical University, Nanning, Guangxi China; 6 Department of Otolaryngology, the Fourth Affiliated Hospital of Guangxi Medical University, Liuzhou, Guangxi China; 7 Department of Pathology, the Fourth Affiliated Hospital of Guangxi Medical University, Liuzhou, Guangxi China; 8 Experimental Center of Medical Science, Guangxi Medical University, Nanning, Guangxi China; 9 Medical Science Laboratory, the First Affiliated Hospital of Guangxi Medical University, Nanning, Guangxi China; 10 Department of Gastroenterology, the First People’s Hospital of Neijiang City, Neijiang, Sichuan China; 11 Department of Hematology, the Fourth Affiliated Hospital of Guangxi Medical University, Liuzhou, Guangxi China

**Keywords:** LINC00930, RBBP5/GCN5, PFKFB3, Glycolysis, Nasopharyngeal carcinoma

## Abstract

**Background:**

Metabolic reprogramming is a hallmark of cancer. However, the roles of long noncoding RNAs (lncRNAs) in cancer metabolism, especially glucose metabolism remain largely unknown.

**Results:**

In this study, we identified and functionally characterized a novel metabolism-related lncRNA, LINC00930, which was upregulated and associated with tumorigenesis, lymphatic invasion, metastasis, and poor prognosis in nasopharyngeal carcinoma (NPC). Functionally, LINC00930 was required for increased glycolysis activity and cell proliferation in multiple NPC models in vitro and in vivo. Mechanistically, LINC00930 served as a scaffold to recruit the RBBP5 and GCN5 complex to the PFKFB3 promoter and increased H3K4 trimethylation and H3K9 acetylation levels in the PFKFB3 promoter region, which epigenetically transactivating PFKFB3, and thus promoting glycolytic flux and cell cycle progression. Clinically, targeting LINC00930 and PFKFB3 in combination with radiotherapy induced tumor regression.

**Conclusions:**

Collectively, LINC00930 is mechanistically, functionally and clinically oncogenic in NPC. Targeting LINC00930 and its pathway may be meaningful for treating patients with NPC.

**Supplementary Information:**

The online version contains supplementary material available at 10.1186/s13046-022-02282-9.

## Background

Nasopharyngeal carcinoma is a rare type of head and neck cancer originates from the nasopharyngeal epithelium, with the highest incidence in southeast Asia [1, 2]. Radiotherapy is the standard treatment for early disease, and radiotherapy combined with chemotherapy is the standard treatment for advanced NPC patients [3]. Cell proliferation and tumor growth for NPC patients is generally acknowledged as the main reason for treatment failure [1, 3, 4]. Therefore, more efforts are required for the development of novel biomarkers and targets for NPC diagnosis and therapy.

Glycolysis, which is also known as the Warburg effect, has been widely recognized as a central hallmark of human cancer [5, 6]. The high frequency of high glycolysis rates in cancer cells remains an established feature of many human tumors. This energy production process provides metabolites for cancer cells and can be used as precursors of anabolic pathway, which supports the biosynthetic needs of malignant cell proliferation [6–8]. A better understanding of the mechanistic links between glycolysis and cell proliferation may ultimately lead to better treatments for human cancer. Phosphofructo-2-kinase/fructose-2,6-biphosphatase 3 (PFKFB3) is a glycolysis-regulatory enzyme, which efficiently catalyzes the production of F-2,6-BP and lactate [9]. Recent studies revealed the unexpected role of PFKFB3 in promoting cell proliferation by regulating the expression of key cell cycle-related proteins [10, 11]. Therefore, PFKFB3 inhibition may be a promising approach for cancer treatment.

Long non-coding RNAs (lncRNAs) are a class of RNA molecules consisting of more than 200 nucleotides without protein-coding potential [12, 13]. LncRNAs are now recognized to constitute a regulatory system that function at the transcriptional and post-transcriptional levels [12, 14, 15]. Recently, many lncRNAs have been identified to promote tumorigenesis through metabolic reprogramming, but the mechanisms remain elusive [16, 17]. Thus, the roles of lncRNAs in metabolism reprogramming and the underlying mechanisms have attracted our interest.

In this study, we identify and functionally characterize a novel metabolism-related lncRNA in NPC. We demonstrate a strong relationship between LINC00930 dysregulation and NPC development. Functionally, LINC00930 has a pivotal role in glucose metabolism remodeling and cell proliferation. We provide evidence that LINC00930 serves as a molecular scaffold for the interaction of RBBP5 and GCN5, thus enhancing H3K4 trimethylation and H3K9 acetylation levels, which transactivates the target gene PFKFB3 in NPC. Therefore, our study reveals a previously unappreciated lncRNA, which connects the glycolytic remodeling and NPC progression and is a promising therapeutic target.

## Methods

### Patient specimens

For cohort 1 (Supplementary Table S1), 71 cases of freshly-frozen tumor tissues and corresponding normal nasopharyngeal epithelium samples were obtained from NPC patients. For cohort 2 (Supplementary Table S2), another 128 paraffin-embedded NPC biopsy tissues and adjacent tissues were collected. Patients of both cohort 1 and 2 were obtained from the Fourth Affiliated Hospital of Guangxi Medical University from January 2006 to December 2017 and had detailed clinical characteristics and long-term follow-up data. All patients were pathologically diagnosed with primary NPC, without receiving any antineoplastic therapy prior to biopsy.

A total of 120 nasopharyngeal brushing samples including normal nasopharyngeal epithelium and pre-cancerous lesions (SM or DYS) were archived and collected from August 2016 to December 2017 in the Fourth Affiliated Hospital of Guangxi Medical University. No corticosteroid, rhinitis spray or NSAIDs should be used within 4 weeks before sampling.

### Cell lines

Eleven NPC cell lines (6-10B, 5-8F, HNE1, HNE2, HNE3, CNE1, CNE2, HONE1, SUNE1, C666–1 and HK-1) were maintained in RPMI-1640 (Invitrogen, Carlsbad, USA) supplemented with 10% fetal bovine serum (FBS, Gibco, Grand Island, USA). The human immortalized nasopharyngeal epithelial cell line (NP69) was cultured in keratinocyte/serum-free medium (Invitrogen) supplemented with bovine pituitary extract (BD Bioscience, CA, USA). All cell lines were genotyped for identity by GENEWIZ Biotechnology Co., Ltd. (Suzhou, China) and tested routinely for Mycoplasma contamination (Yeasen, # 40601ES20).

### Reagents and antibodies

Trichostatin A (#CSN12139), Anacardic Acid (#CSN13640) and Curcumin (#CSN19424) were purchased from CSNPharm. YF-2 (#S0022) and PFK15 (#S7289) were obtained from Selleck chemicals. Antisense oligonucleotide (ASO) of LINC00930 was provided from Takara Bio. Antibodies against p27 (#sc1641) was obtained from Santa cruz Biotechnology. Antibodies against CDK1 (#ab133327), PFKFB3 (#ab181861), RBBP5 (#ab154755), GCN5 (#ab217876) and β-tubulin (#ab6046) were purchased from Abcam. Antibodies against H3K4me3 (#9751), H3K9me3 (#13969), H3K27me3 (#9733) IgG (#2985) were provided by Cell Signaling Technology.

### Plasmid constructs, lentivirus and siRNAs

The full-length cDNA of human LINC00930 was PCR-amplified and subcloned into the pCDNA3.1 vector (Invitrogen), and the final construct was verified by sequencing. The empty pcDNA3.1 vector was used as the control. The small hairpin RNA (shRNA) of the LINC00930 was provided by Addgene. The siRNAs targeting human LINC00930, PFKFB3, RBBP5, GCN5 and the scramble siRNA control were all purchased from Takara (Dalian, China). Details of shRNAs and siRNAs target sequences are listed in Supplementary Table S3. N-Terminal FLAG-tagged RBBP5 and HA-tagged GCN5 expression vectors (for expression in mammalian cells) were provided by OBiO Technology (Shanghai, China). Point mutations (Supplementary Table S4) in constructs were generated using a Site-Directed Mutagenesis Kit (Agilent). Plasmid vectors and siRNAs transfection was conducted using the Lipofectamine 3000 reagent (Life Technologies) according to manufacturer’s instructions. Cells were harvested 48–72 h post transfection for various assays.

### Differentially expressed lncRNAs analysis

We profiled differentially expressed genes between NPC and normal tissue using two independent sets of microarray data, GSE64634 [18] and GSE12452 [19]. Both microarray data were performed and analysed based on Affymetrix Human Genome U133 Plus 2.0 platform. GSE64634 has 12 NPC and 4 normal nasopharyngeal samples; GSE12452 contains 31 NPC and 10 normal nasopharyngeal samples, respectively. GSE64634 and GSE12452 gene expression profiles date was analyzed by means of DESeq2 online software with fold change ≥2.0 and the false discovery ratio (FDR) < 0.01. Four hundred twenty-one and three hundred eighty-two differentially expressed genes were obtained in GSE64634 dataset GSE12452 dataset, respectively. The differentially expressed lncRNAs between NPC and non-tumor tissues were listed in Supplementary Table S5.

### In situ hybridization for LINC00930

We used three 30-base nucleotide probes from different regions of the LINC00930 corresponding to bp 550–579, 875–904 and 1211–1240 of the LINC00930. The sequences were 5′-CTC TTC ATT CAA CTG GTA CCT CAG TTG GAA-3′, 5′-TGT GAG GAC AAC TGG AAG GTG CCC CTC ACC-3′, and 5′-CTG AGG GGT CAC CAG TCA CAG CCA TGG CCT-3′. The probes were synthetized and labeled with DIG-dUTP at the 3′ end using a kit from Boster (# MK10098, Wuhan, China). In situ hybridization was performed as previously described [20]. Briefly, After deparaffinized in xylene and re-hydrated in gradient ethanol solution, the slides were then fixed in 4% paraformaldehyde. After that, the slides were incubated with pre-hybridizing solution at 37 °C for 2 h, washed with PBS for 3 times, and incubated with specific targeted probes dissolved in sheared salmon sperm DNA (Invitrogen) and yeast tRNA solution, incubated at 4 °C overnight. The concentration of probe solution was 500 ng/mL. The slides were then washed in 37 °C gradient SSC solution. Finally, dig nucleic acid detection kit (Roche) was used for immunological detection of digoxin. In order to avoid the degradation of RNA by RNase, all glassware and solutions involved in ISH assay should be treated with RNase inhibitor DEPC.

A quantitative scoring criterion for in situ hybridization was used in which both the staining intensity and the number of positive cells were recorded as previously described [21]. Specifically, the staining scores of LINC00930 was double-blind read by two experienced pathologists. LINC00930 was mainly located in the nucleus. The positive cell percentage and staining intensity of positive cells were scored respectively. Positive cell percentage evaluating criteria: Five high-power visual fields were observed on each slice, and the percentage of positive cells was counted. Less than 5% was 0, 5% ~ 25% was 1, 26% ~ 50% was 2, 51% ~ 75% was 3, and 76% ~ 100% was 4. Positive staining intensity evaluating criteria: colorless is 0, light yellow is 1, brownish yellow is 2 and dark brown is 3. The staining score was obtained by multiplying the two scores: 0–6 points were considered as low expression, 7–12 points were considered as high expression. Correlations between different clinical status and LINC00930 positive expression were analyzed using a chi-square test.

### RNA extraction, reverse transcription, and quantitative PCR

TRIzol reagent (Invitrogen) was utilized to isolate total RNA from NPC tissues and cells. The RNeasy serum/plasma kit (QIAGEN GmbH) was applied to extract total RNA from serum samples. The RNA quality and amount were evaluated by a NanoDrop 3300 spectrophotometer (Thermo Scientific). Reverse transcriptase (Promega) was used to perform reverse transcription. SYBR Green qPCR Super Mix-UDG (Thermo Fisher) was employed to conduct Quantitative real-time PCR (qRT-PCR). β-tubulin was utilized as the normalization control. Specific primers are shown in Supplementary Table S4.

### Colony formation and CCK-8 assays

For the colony formation assay, 1000 cells in 2 ml medium were seeded into six-well plates and cultured for 6 or 11 days. Cell colonies were then successively fixed, stained, and counted. For the CCK-8 assay, 2 × 10^3^ cells in 200 μl medium solution were cultured in 96-well plates. Then, a 20 μl CCK-8 reagent (DOJINDO) was added to each well. After the 96-well plates were incubated for 2–4 h at 37, we then measure the absorbance at 450 nm for each sample well.

### Flow cytometry analysis

Flow cytometry analysis was performed as previously described [22, 23]. In brief, 5 × 10^5^ cells were plated into 6-well plates. After adhering to the well, the cells were treated with serum starvation for 24 h and harvested by trypsin after releasing for 48 h. Cells were trypsinized, washed three times with PBS, and fixed overnight in 75% pre-cooled ethanol at − 20 °C. Cells were washed three times with PBS, and PI/RNA reagent (BD, USA) was added and incubated for 15 min at room temperature in the dark. Single-cell suspension was obtained through a nylon membrane and detected by flow cytometer. The results were analyzed by Modfit 3.2 software. Three independent experiments were performed.

### Metabolic assays

Seahorse Biosciences XF96 analyzer (North Billerica, MA, USA) was applied to determine ECAR (extracellular acidification rate) and OCR (oxygen consumption rate), as previously described [15, 24]. Cells transfected with control siRNA, LINC00930 shRNA, empty vector, and LINC00930 overexpressing vector, were seeded in a XF96-well Assay plates and incubated overnight. ECAR was measured under basal conditions and in response to 10 mM glucose, 5 μM oligomycin, and 100 mM 2-deoxyglucose (all from Sigma-Aldrich). OCR was measured under basal conditions and in response to 5 μM oligomycin, and 50 mM FCCP and 20 mM Rota/AA (all from Sigma-Aldrich). For evaluation of the real-time ECAR and OCR, 3 min of mixture, 3 min of waiting, and 3 min of measurement was performed in turn. ECAR and OCR measurements were normalized to total protein content and reported as mpH/min.

### Glucose metabolic flux analysis

^13^C-Labeled intracellular metabolites were detected as previously described [24]. Briefly, cells (2 × 10^7^) were incubated with 2 g/L ^13^C6-labeled glucose (Sigma-Aldrich, St. Louis, MO) for 2 h. Metabolites were extracted, and those including at least one ^13^C atom were analyzed using an LCsystem equipped with a TripleTOF 5600 mass spectrometer (SCIEX, Framingham, MA, USA). The concentrations of ^13^C6-labeled metabolites were normalized to cell number.

### F-2,6-BP level measurement

NPC cells and xenograft tumor tissues were collected to detect F-2,6-BP levels using a coupled enzyme reaction as previously reported [25]. Briefly, Cells and tissues samples were homogenized and lysed in NaOH and then heated for 10 min at 80 °C. After cooling, the samples were centrifuged and the supernatant neutralized with acetic acid. F-2,6-BP was measured by the stimulation of PP_i_:PFK and assayed in the presence of 0.5 mM pyrophosphate and 1 mM fructose 6-phosphate. Finally, the F-2,6-BP concentration was normalized to total cellular protein according to the manufacturer’s instructions.

### Lactate concentration detection

Lactate concentration in growth medium and xenograft tumor was measured using a Lactate Assay kit (Abcam) according to the manufacturer’s protocols. First, xenograft tumor tissues were powdered and homogenized in absolute methanol to obtalin tissue homogenate. Then, the cell medium or tissue homogenate was collected and then incubated with reaction mix at room temperature for 30 min. Absorbance at a wavelength of 450 nm was measured using a microplate reader (Bio-Tek). Lactate production = lactate in cultured medium (mM) − lactate in the fresh medium (mM).

### PFK activity

PFK activity in of NPC cells was determined as reported before [26]. Cells were lysated in the basic buffer containing 50 mM Tris-HCl (pH = 7.4), 5 mM MgCl_2_, 5 mM (NH_4_)_2_SO_4_, 1 mM F-6-P, 1 mM ATP, 0.5 mM NADH, 2 mU/ml aldolase, 2 mU/ml triosephosphate isomerase, 2 mU/ml α-glycerophosphate dehydrogenase. PFK activity was assayed in the presence increasing concentrations of F-2,6-BP (1 μM, 10 μM, and 50 μM) or AMP (1 μM, 2 μM, 10 μM, 50 μM, and 150 μM) added to buffer. Enzymatic activity was measured in triplicate spectrophotometrically at 340 nm.

### RNA pull-down assay

Biotinylated lncRNAs were refolded in NEB enzyme buffer with RNaseOUT (Invitrogen). To prepare cell lysates, NPC cells were harvested into RNA pull-down buffer containing 0.25% NP-40, 10 mM Tris-HCl (pH = 7.0), 1.5 mM MgCl_2_, 10 mM KCl, 0.5 mM DTT, 1 mM PMSF, and protease inhibitor complex (PIC). Cell suspension was gently pipetted up and down 15 times and then centrifuged at 12000 rpm for 30 s immediately. The pellets were dissolved in 800 μl of RNA pull-down buffer as the nuclear protein. For the pull-down incubations, nuclear lysates were precleared with streptavidin beads and then incubated with 2 μg of biotinylated RNA and 40 μl of streptavidin beads for 4 h at 4 °C. Beads were collected by centrifugation and RNA-associated proteins were eluted and subjected to MS analysis or Western blotting.

### RNA immunoprecipitation (RIP)

RIP assay was conducted with a Magna RNA-binding protein immunoprecipitation kit (Millipore, Bedford, MA) as previously described [24]. Briefly, cells were crosslinked with formaldehyde and then lysed with RIP buffer. Then cell lysate was incubated with protein beads and antibody complex overnight at 4 °C. Anti-RBBP5 antibody (1:100, Abcam), anti-GCN5 antibody (1:50, Abcam) and negative control IgG (1:100, Cell Signaling Technology) were used in this study. RNA samples were extracted and subjected to Northern blot and qRT-PCR analysis. RNA levels were normalized to the input (20%).

### Co-immunoprecipitation (co-IP)

Co-immunoprecipitation (Co-IP) was performed as previously described [27]. Briefly, cells were lysed in 1 mL of IP lysis buffer (50 mM Tris-HCl at pH 7.4, 1 mM EDTA at pH 8.0, 150 mM NaCl, 1% NP-40, PIC, PhosSTOP, and PMSF). Cell lysates were immunoprecipitated with indicated primary antibodies overnight at 4 °C and then Protein A/G (5:1) for 4 h. The beads were washed three times with the lysis buffer and eluted in SDS sample buffer. The eluted immunocomplexes were resolved by SDS-PAGE, followed by Western blotting.

### Chromatin immunoprecipitation (ChIP) and chromatin isolation by RNA purification (ChIRP)

ChIP assay was employed to determine the binding of the chromatin-modifying complexes to the promoter regions of PFKFB3. Cells were fixed and immunoprecipitated using the EZ-Magna ChIP assay kit as recommended by the manufacturer (Millipore, USA). Antibodies anti-H3K4me3, anti-H3K9me3 and anti-H3K27me3 were utilized to immunoprecipitate purified chromatin. Primers used to amplify promoter regions of PFKFB3 are shown in Supplementary Table S4. For ChIRP assay, a 3′ end Biotin-TEG modified-DNA probe targeting LINC00930 was synthesized and purified by HPLC. Probes were divided into the odd number group (1, 3, 5, etc.) and the even number group (2, 4, 6, etc.), and probes targeting LacZ were selected as nonspecific controls.

### Western blot

Total protein was extracted from cultured cells by using RIPA lysis buffer supplemented with 1% proteinase inhibitor complex (PIC, Roche) and 1% PhosStop (Roche). Proteins were separated by 10% SDS-PAGE gel electrophoresis and then transferred to PVDF membranes (Millipore). The membranes were blocked with 5% BSA for 1 h and then incubated with primary antibodies and horseradish peroxidase–conjugated secondary antibodies successively, and the signals were detected with ECL Substrate Kit (Thermo Scientific).

### Immunohistochemistry (IHC)

IHC was performed using an EnVision HRP kit (Dako) as previously described [22]. Briefly, paraffin-embedded mouse and human tissues were deparaffinized in xylene, re-hydrated in gradient ethanol. Then, antigen was retrieved by boiling the sample for 60 min. Samples were incubated with anti-PFKFB3 antibody at 4 °C overnight. The sections were stained with secondary antibody for 30 min at room temperature and then stained DAB reagent. The staining score of PFKFB3 was obtained by multiplying the the percentage and staining intensity of positive cells. The results were scored by two independent investigators. A score of 0–6 was considered to represent low expression and a score of 7–12 was considered to represent high expression.

### Subcellular fractionation

A Cytoplasmic & Nuclear RNA Purification Kit (Norgen Biotek Corp, Canada) was used to detect LINC00930 expression in cytoplasmic and nuclear fractions. According to the manufacturer’s instructions, RNA was extracted from the cytoplasmic and nuclear fractions and subjected to qRT-PCR. β-Actin (ACTB) and GAPDH were applied as a cytoplasmic marker, and U1 was used as a nuclear marker.

### Luciferase reporter assay

Different promoter regions of PFKFB3 (Supplementary Table S4) was amplified and cloned into pGL3 basic luciferase reporter vectors. NPC cells were grown and transfected with vectors of promoter-firefly LUC, internal Renilla LUC and other relevant siRNAs. Forty-eight hours post-transfection, cells were washed with PBS. The luciferase reporter assay was conducted using a Dual-Luciferase Reporter Assay System (Promega, E1910) according to the manufacturer’s instructions. Luminescence was measured using a Gen5 microplate reader (BIOTEK, USA).

### Xenografts

For cell-derived xenograft (CDX), NPC cells (2 × 10^6^) were injected subcutaneously into the dorsal flanks of 4-week-old female BALB/c nu/nu mice (8 mice per group). Tumor volume was measured at the indicated time points and calculated as length × width^2^/2. For patient-derived xenograft (PDX), fresh NPC tumor samples were immediately inoculated subcutaneously into both flanks of nude mice. When the successfully established PDXs (F1) reached ~ 500 mm^3^, the tumors were transplanted to other mice (F2). Eventually, the mice bearing F3 grafts were used for radiotherapy and chemotherapy experiments.

### Drugs treatment

For in vitro cell culture model, LINC00930 inhibitor (ASO LINC, Takara) and PFKFB3 inhibitor (PFK15, Selleck) was dissolved in physiological saline buffer and DMSO, respectively. Five thousand cells per well were seeded in a 96-well plate and cultured for 24 h. ASO LINC or/and PFK15 were added to indicated concentrations. Then, cells were subjected to different irradiation intensity (0, 2, 4, 6 and 8 Gy) with 6 MV X-rays and cultured for another 48 h. Relative survival was calculated as absorbance of the drug-treated cells/absorbance of the corresponding cells without drug treatment × 100%.

For xenograft animal models, PFK15 was dissolved in the solvent solution containing 5% DMSO, 40% PEG-300, 5% Tween-80, 5% Propylene glycol and 45% H_2_O (50 mg/kg, intraperitoneal administration). In vivo-optimized LINC00930 inhibitor (ASO LINC) was dissolved in physiological saline buffer (5 nM per injection, intratumoral administration). Once tumor sizes reached 100–150 mm^3^, mice were randomly assigned into five groups (8 mice per group), including vehicle group, vehicle + radiotherapy group, LINC00930 inhibitor + radiotherapy group, PFKFB3 inhibitor + radiotherapy group, ASO LINC + PFK15 + radiotherapy group. Mice for radiotherapy treatment were exposed to irradiation by 6 MV X-ray, with 2 Gy per day, twice a week. After 4 weeks, all mice were sacrificed, and the tumor weights were measured and calculated as length×width^2^/2. The animal experimental protocols were approved by the Medical Experimental Animal Care Commission of Guangxi medical university and Jining Medical University, and performed in accordance with the institutional ethical guidelines for animal experiments.

### Statistical analysis

Statistical analysis was carried out using SPSS 19.0 software and GraphPad Prism 5 and Image J software. Two-tailed and unpaired Student’s t-tests were used for two group comparisons. One-way ANOVA tests were applied for multiple groups comparisons. Wilcoxon test and nonparametric Mann-Whitney-Wilcoxon test were both utilized for evaluating the differences of tumor tissues and paired controls. Pearson correlation analysis was performed to analyze the correlation of two molecules. Survival curves were estimated using the Kaplan-Meier method and compared using the log-rank test. Data are shown as the mean ± SD. *P* < 0.05 was considered statistically significant.

## Results

### LINC00930 is a metabolism-related lncRNA and clinically relevant with the progression of NPC

**Fig. 1:**
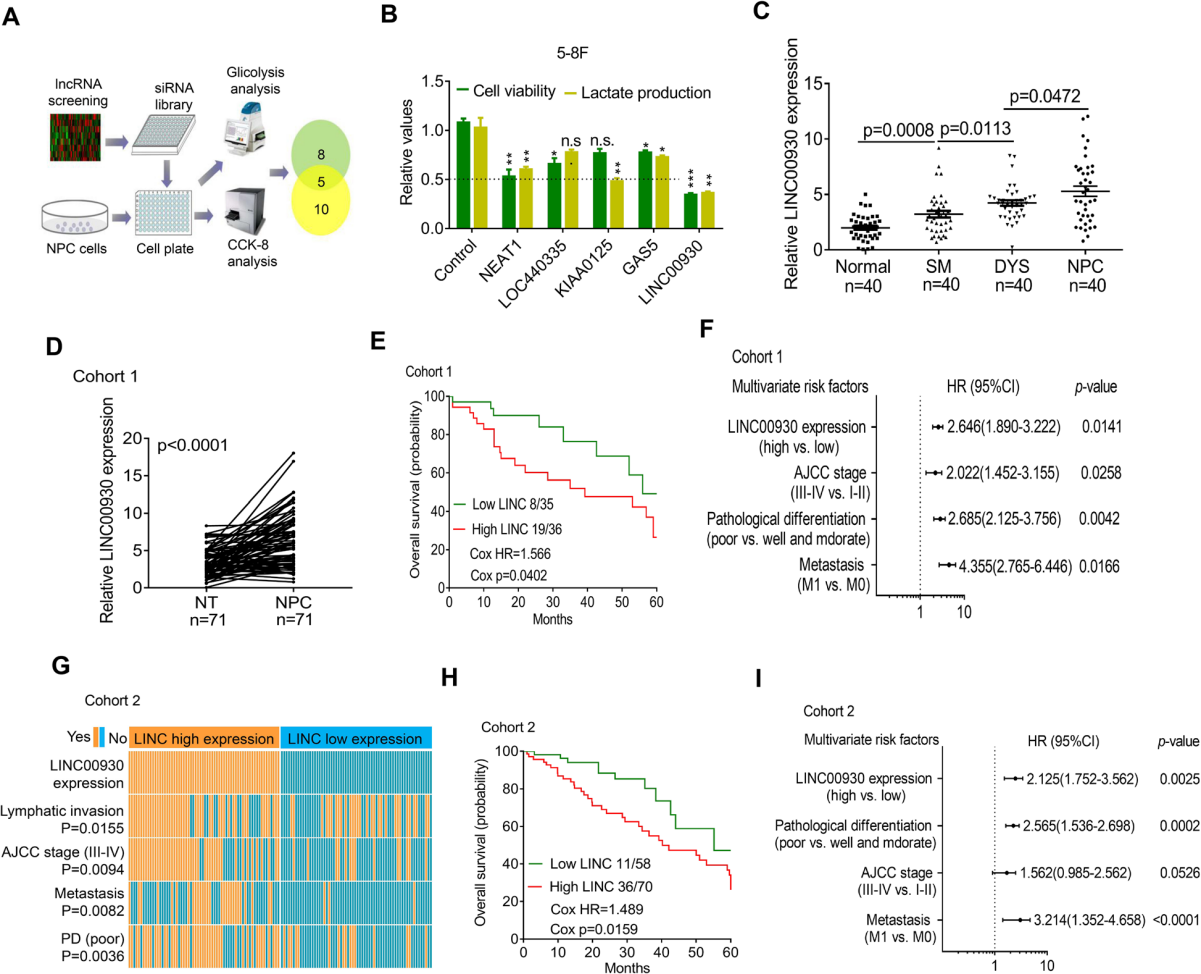
LINC00930 is a metabolism-related lncRNA and clinically relevant with the progression of NPC. **a** Experimental scheme for identifying lncRNAs potentially involved in both cell viability and glucose metabolism in NPC. **b** Five lncRNAs regulated both cell proliferation and lactate production in 5-8F cell (*n* = 3 biologically independent samples). **c** Statistical analysis of LINC00930 expression in normal, SM, DYS, and NPC tissues. (*n* = 40, nonparametric Mann-Whitney test). SM, squamous metaplasia; DYS, dysplasia. **d** LINC00930 expression were measured in NPC tissues and their adjacent tissues in cohort 1 (*n* = 71, wilcoxon test). **e** Survival was analyzed and compared between patients with low and high levels of LINC00930 in cohort 1 (*n* = 71, log-rank test, two-sided). LINC, LINC00930; HR, Hazard Ratio. **f** Multivariable analysis was performed in cohort 1. All the bars correspond to 95% confidence intervals. **g** Comparing different AJCC stages, positive or negative lymphatic invasion, metastasis, and pathological differentiation between LINC00930 high- and low-expression tumors of cohort 2. The heatmap illustrated the association of different clinical characters with LINC00930 high- and low-expression tumors using ISH analysis in cohort 2 (*n* = 128, chi-square test). PD, pathological differentiation. **h** Survival was analyzed and compared between patients with high and low levels of LINC00930 expression in tumor in cohort 2 (*n* = 128, log-rank test, two-sided). **i** Multivariable analysis was performed in cohort 2. All the bars correspond to 95% confidence intervals

To find oncogenic lncRNAs that significantly affect NPC development, we processed and analyzed RNA sequencing (RNA-seq) expression data from two independent NPC studies (Fig. S1a). We first used DESeq2 program to obtain a list of differentially expressed transcripts (cancer tissues vs. normal tissues) from each cohort and then used metaRNAseq to perform meta-analysis on the two lists. The combined *p*-value from multiple testing was further corrected using a false discovery rate (FDR) < 0.01. Eventually, we identified 96 clinically relevant lncRNAs in NPC (Supplementary Table S5). To narrow down candidate lncRNA, siRNA-based high-throughput screen (targeting top 50 lncRNAs) was applied in NPC cells. Cell viability and lactate production served as indexes to indicate cell proliferation and glycolysis level. As shown in Fig. 1a, 10 lncRNAs were involved in cell proliferation regulation and 8 lncRNAs were involved in glycolysis regulation. Among 5 overlapped lncRNAs, LINC00930 attracted our attention because the strong regulation of cell proliferation and glycolysis in both cell (Fig. 1b and Fig. S1b). Furthermore, real-time PCR showed that LINC00930 expression gradually increased from normal nasopharyngeal epithelium (NPE) to squamous metaplasia (SM), to dysplasia (DYS) and to NPC (Fig. 1c). Thus, an increase in LINC00930 is an important event in the multistep progression of nasopharyngeal carcinogenesis.

Bioinformatics analysis revealed that LINC00930 was located on chromosome 15q26.1 and had 4 exons. Then, we evaluated the coding potential of LINC00930 by examining the DNA sequence using the Coding Potential Calculator (http://cpc.cbi.pku.edu.cn/) [28]. A score of − 1.125 was obtained for LINC00930 suggesting the coding potential of LINC00930 was very low. Similarly, when we examined the coding potential of MALAT1, a well-known lncRNA, we obtained a score of − 1.032. In contrast, we obtained a score of 14.226 for β-tubulin. Moreover, in vitro translation assays (Fig. S1c) also confirmed LINC00930 has no coding capability.

To determine the pathological and clinical significance of LINC00930 expression in NPC, we analyzed the expression of LINC00930 in 71 cases of fresh NPC and adjacent tissues (cohort 1, Supplementary Table S1). Real-time PCR revealed that LINC00930 was significantly increased in cancer versus adjacent tissues of cohort 1 (Fig. 1d). Further, high level of LINC00930 was closely related to advanced NPC TNM stages (Fig. S1d). We next analyzed the correlation between the LINC00930 and the clinical outcome in cohort 1. The Kaplan-Meier analyses showed that high expression of LINC00930 was significantly associated with a poor prognosis in these patients (Fig. 1e). Multivariate regression analyses of cohort 1 demonstrated that LINC00930 expression was an independent predictor of NPC aggressiveness with significant HRs for predicting clinical outcome. Its predictive value was comparable to that of the pathological differentiation pattern (Fig. 1f).

To further validate the correlation of LINC00930 with NPC progression, we detected and compared LINC00930 expression by in situ hybridization (ISH) in an additional 128 paraffin-embedded NPC and adjacent tissues (cohort 2, Supplementary Table S2, Fig. S1e). Consistent with the results in cohort 1, we found that LINC00930 expression positively correlated with lymphatic invasion, AJCC stage, metastasis, and pathological differentiation (Fig. 1g). Moreover, the Kaplan-Meier analyses showed that high expression of LINC00930 indicated an unfavourable prognosis (Fig. 1h) in multivariate regression analyses (Fig. 1i).

We next analyzed the correlation between LINC00930 and the clinical outcome based on external database. First, normalized expression level of LINC00930 was significantly increased in cancer tissues compared to adjacent non-tumor tissues from the TCGA HNSC dataset (Fig. S1f). Consistently, high LINC00930 levels indicated an poor overall survival in TCGA HNSC cohort (Fig. S1g and S1h). We next validated LINC00930 expression in another two cohort of NPC samples from the GEO database repository (GSE118719, Fig. S1i; GSE40290, Fig. S1j). Additionally, high LINC00930 expression was associated with clinicopathological parameters in the GSE12452 dataset, including advanced AJCC stages (Fig. S1k). Collectively, LINC00930 is a metabolism-related lncRNA and clinically relevant with the progression of NPC.

### LINC00930 promotes cell proliferation and glycolysis

**Fig. 2:**
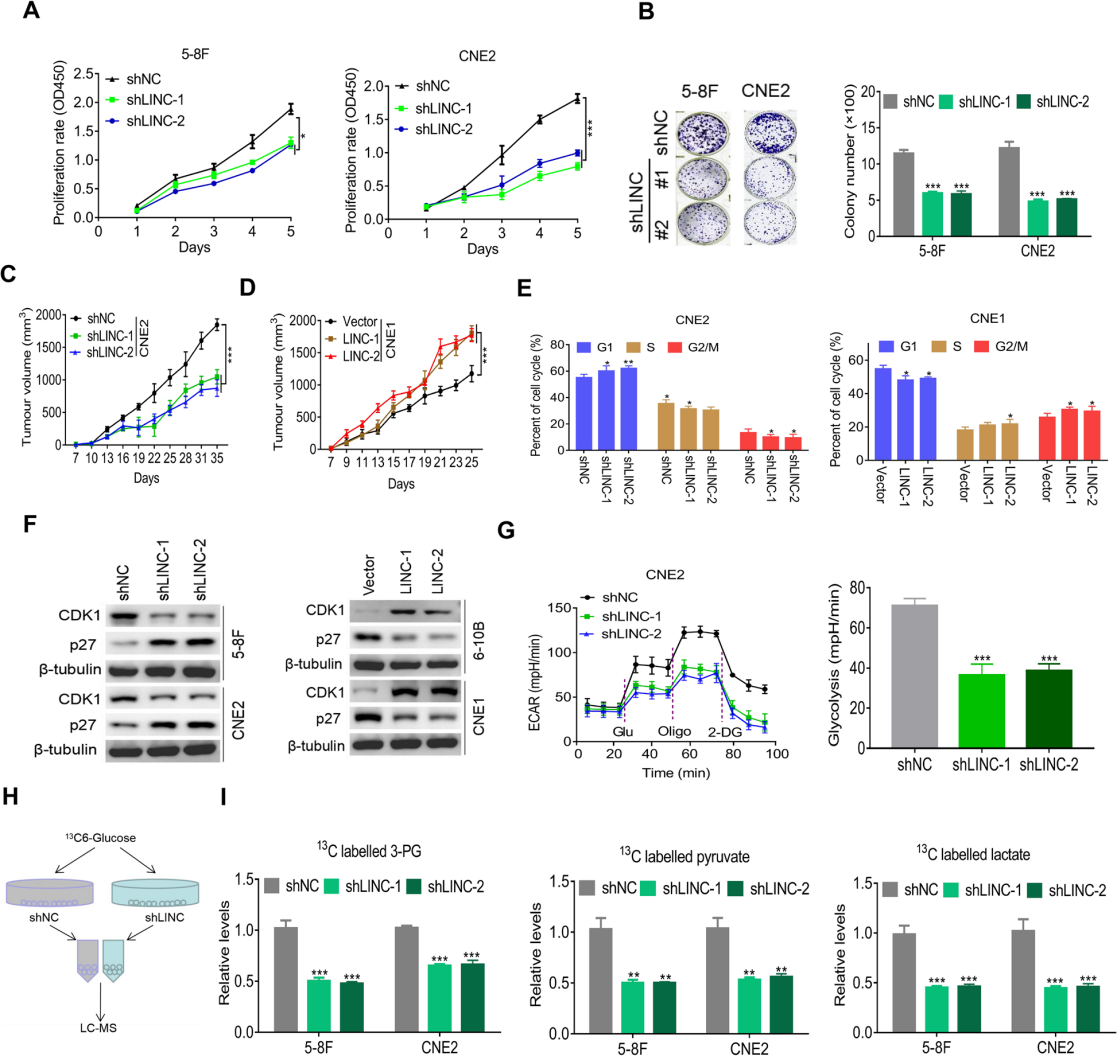
LINC00930 promotes cell proliferation and glycolysis. **a** and **b** Cell viability and colony formation in 5-8F and CNE2 cells stably knockdown LINC00930. **c** and **d** Subcutaneous tumor formation in nude mice. CNE2 cells with LINC00930 knockdown and CNE1 with LINC00930 overexpression were injected into one flank of the mouse. Tumor volume was measured and calculated at the indicated time (*n* = 8). **e** CNE2 cell with LINC00930 knockdown or CNE1 cell with LINC00930 overexpression were collected 48 h after releasing from synchronization with serum starvation. The cell cycle distributions were examined by flow cytometry analysis. DNA content was quantified using Modfit 3.2 software. Quantification of the cell population in each phase is presented. **f** CDK1 and p27 expression levels were detected by western blotting in 5-8F and CNE2 cells with LINC00930 knockdown (left) and in 6-10B and CNE1 cells with LINC00930 overexpression (right). **g** Left: The ECAR was measured in CNE2 cells with LINC00930 knockdown using an XF Extracellular Flux Analyzer. Right: Statistical analysis of the effects of LINC00930 knockdown on glycolytic activity. **h** Flowchart of the experiments for identifying the role of LINC00930 in glucose metabolism. **i** ^13^C-Labeled metabolic intermediates of glycolysis were decreased after LINC00930 knockdown. The *p*-value in **a, c & d** was determined by one-way analysis of variance (ANOVA) with Dunnett’s multiple comparisons test, no adjustments were made for multiple comparisons. The *p*-value in **b, e, g** & **i** was determined by a two-tailed unpaired Student’s *t* test. * *p* < 0.05; ** *p* < 0.01; *** *p* < 0.001

Because LINC00930 is potentially involved in cell proliferation and lactate production, we further investigated the functional role of LINC00930 in cellular behaviors. We first examined LINC00930 expression in immortalized normal nasopharyngeal epithelial NP69 cell and a series of NPC cell lines (Fig. S2a). Then, we stably established LINC00930-knockdown cell lines using 5-8F and CNE2 cells and LINC00930-overexpressing cell lines using 6-10B and CNE1 cells (Fig. S2b). As expected, cell proliferation and colony formation were dramatically reduced upon LINC00930 knockdown (Fig. 2a and b). Conversely, overexpression of LINC00930 substantially enhanced cell proliferation rate and colony formation ability (Fig. S2c and S2d). The oncogenic role on LINC00930 was confirmed in xenograft models. As shown in Fig. 2c, d and S2e, knockdown of LINC00930 significantly suppressed tumor growth, while LINC00930 overexpression promoted tumor growth, as demonstrated by the xenograft tumor growth curve. Finally, we detected LINC0093 expression in xenograft tumors and confirmed altered LINC00930 expression in different models (Fig. S2f). Altogether, LINC00930 is functionally oncogenic in NPC.

We further evaluated the effect of LINC00930 on the cell cycle distribution. NPC cells were synchronized with serum starvation for 24 h. After release for 48 h, flow cytometry analysis showed that the percentage of cells at G1 phase was significantly higher in LINC00930-knockdown CNE2 cells and lower in LINC00930-overexpressing CNE1 cells compared to their corresponding control cells (Fig. 2e and Fig. S2g). CDK1/p27 are critical regulators of cell cycle progression. As shown in Fig. 2f and Fig. S2h, CDK1 was downregulated in LINC00930 knockdown NPC cells; whereas p27, the CDK inhibitor, was upregulated upon LINC00930 knockdown, and vice versa. The result suggests that CDK1/p27 may be involved in the process of LINC00930-induced cell proliferation regulation.

Given that LINC00930 was a metabolism-related lncRNA, ECAR and OCR levels were assayed. We demonstrated that LINC00930 knockdown significantly impaired glycolysis (Fig. 2g). Reciprocally, ectopic LINC00930 expression increased the glycolytic capacity compared with control group (Fig. S2i). Moreover, OCR value was slightly decreased in LINC00930 knockdown CNE2 cell (Fig. S2j). ^13^C-labeled metabolome detection analyzes the ^13^C-label status of intracellular intermediate metabolites, so as to systematically quantify the relative size and distribution of each metabolic flux in cells. Here, we found LINC00930 knockdown dramatically reduced glycolysis metabolites, including 3-phosphoglycerate, pyruvate, and lactate in NPC cells after incubation with ^13^C6-glucose for 2 h (Fig. 2h-i), confirming LINC00930 facilitates glycolytic metabolism in NPC. Collectively, LINC00930 plays a crucial role in promoting cell proliferation and glycolysis of NPC.

### PFKFB3 is a downstream target of LINC00930

**Fig. 3:**
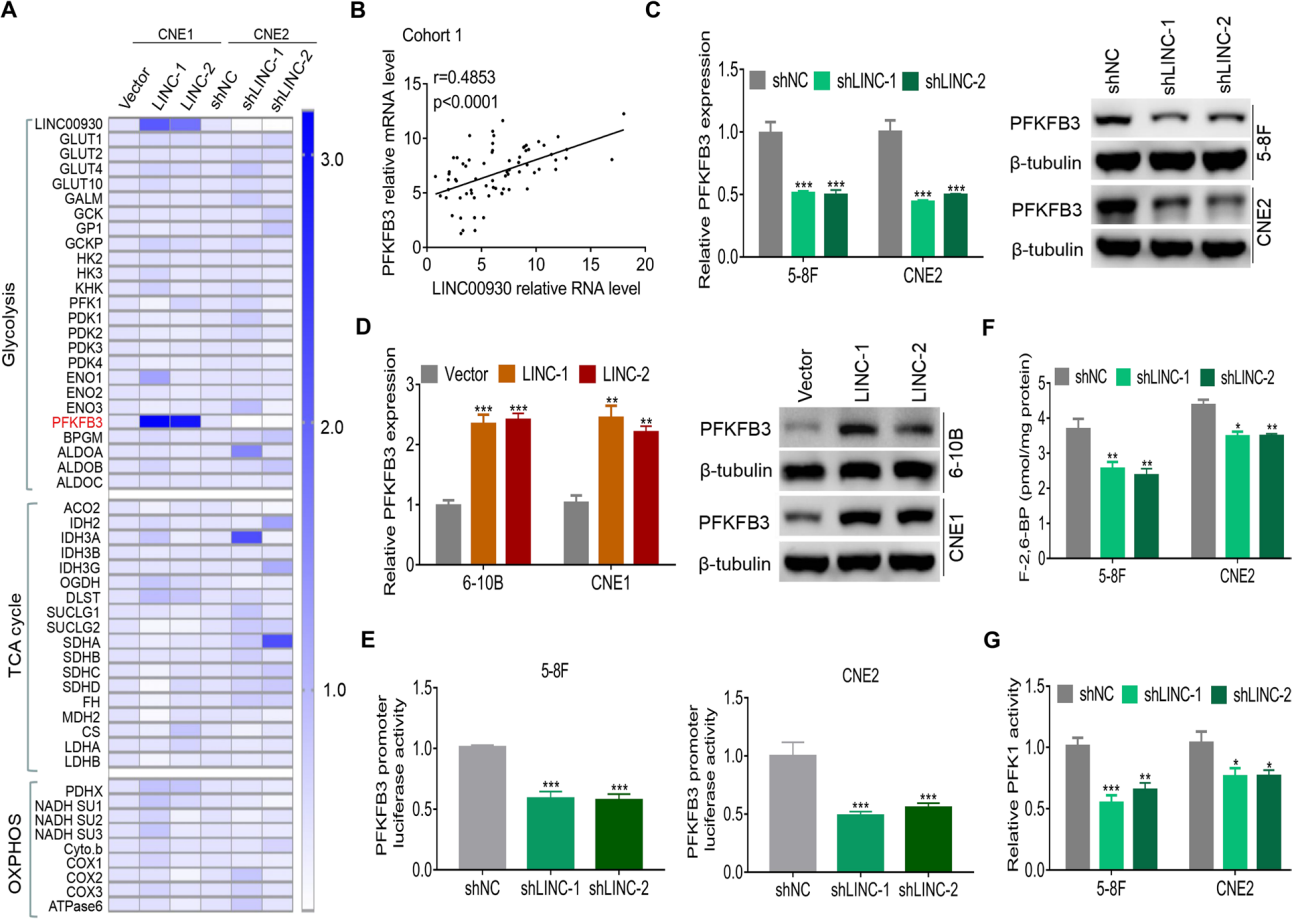
LINC00930 correlates with and regulates PFKFB3. **a** The transcripts of glucose metabolism, TCA and oxidative phosphorylation (OXPHOS)-related genes were detected by real-time PCR in CNE1 cells with LINC00930 overexpression and CNE2 cells with LINC00930 knockdown. **b** The correlation between LINC00930 transcript level and PFKFB3 mRNA level was measured in cohort 1 (*n* = 71, spearman rank-correlation analysis). **c** and **d** The PFKFB3 expression was detected with real-time PCR and western blot in NPC cells with LINC00930 knockdown or LINC00930 overexpression. **e** Luciferase reporter vector was generated by inserting the promoter region (− 2000 bp to + 200 bp) of the PFKFB3 gene. The reporter vectors were then co-transfected into 5-8F and CNE2 cells with LINC00930 or control shRNAs. Cells were harvested for luciferase activity assay. **f** and **g** Intracellular F-2,6-BP level and PFK1 activity were assayed in NPC cells with LINC00930 knockdown. The *p*-value in **c, d, e, f** & **g** was were determined by a two-tailed unpaired Student’s *t* test. * *p* < 0.05; ** *p* < 0.01; *** *p* < 0.001

To clarify the molecular mechanisms underlying LINC00930-mediated glucose metabolism remodeling, we examined transcription of a panel of glucose metabolism-related genes upon LINC00930 knockdown or overexpression. Among these genes, PFKFB3 attracted our attention because of its remarkable change, and its crucial regulatory role in glycolysis (Fig. 3a). PFKFB3 has been reported to be up-regulated and exhibit tumor-promotive roles in several types of malignant tumor [29, 30]. First, we found that PFKFB3 was significantly increased in cancer versus adjacent tissues in cohort 1 (Fig. S3a), cohort 2 (Fig. S3b), TCGA HNSC dataset (Fig. S3c) and two GEO datasets (GSE64634, Fig. S3d; GSE12452, Fig. S3e). Further, the Kaplan-Meier analyses showed that high expression of PFKFB3 indicated an unfavourable prognosis in cohort 1 (Fig. S3f), cohort 2 (Fig. S3g) and TCGA HNSC dataset (Fig. S3h). Next, a positive correlation between LINC00930 and PFKFB3 transcript levels was found in cohort 1 (*r* = 0.4854, *p* < 0.001, Fig. 3b) and in TCGA HNSC dataset (*r* = 0.3107, *p* < 0.001, Fig. S3i). Real-time PCR and western blot analysis confirmed that LINC00930 knockdown markedly decreased the expression level of PFKFB3 (Fig. 3c and S3j), while LINC00930 overexpression upregulated the expression levels of PFKFB3 (Fig. 3d and S3k) in NPC cells. Additionally, we detected and compared PFKFB3 expression by immunohistochemistry (IHC) analysis in xenograft tumors. IHC analysis confirmed that LINC00930 knockdown significantly decreased the expression level of PFKFB3 (Fig. S3l), while LINC00930 overexpression upregulated the expression levels of PFKFB3 (Fig. S3m) in xenograft models. Dual-luciferase reporter assay showed that LINC00930 knockdown dramatically suppressed PFKFB3 promoter activity in 5-8F and CNE2 cells (Fig. 3e). Altogether, the data suggest that LINC00930 promotes the transcription of PFKFB3 in NPC.

PFKFB3 phosphorylates fructose-6-phosphate (F-6-P) to fructose-2,6-bisphosphate (F-2,6-BP), which subsequently allosterically activates phosphofructokinase-1 (PFK1) and stimulates high glycolytic flux in human cancers [31, 32]. Therefore, we examined if LINC00930 regulated F-2,6-BP level and PFK1 activity. As shown in Fig. 3f and g, compared to control cells. F-2,6-BP level and PFK1 activity were both markedly reduced in LINC00930-knockdown NPC cells. Conversely, overexpression of LINC00930 significantly increased F-2,6-BP level and PFK1 activity (Fig. S3n and S3o). Thus, these data suggest that LINC00930 increases F-2,6-BP level by regulating PFKFB3 expression, thereby promoting glycolysis and cell cycle progression.

### LINC00930 interacts with RBBP5 and GCN5

**Fig. 4:**
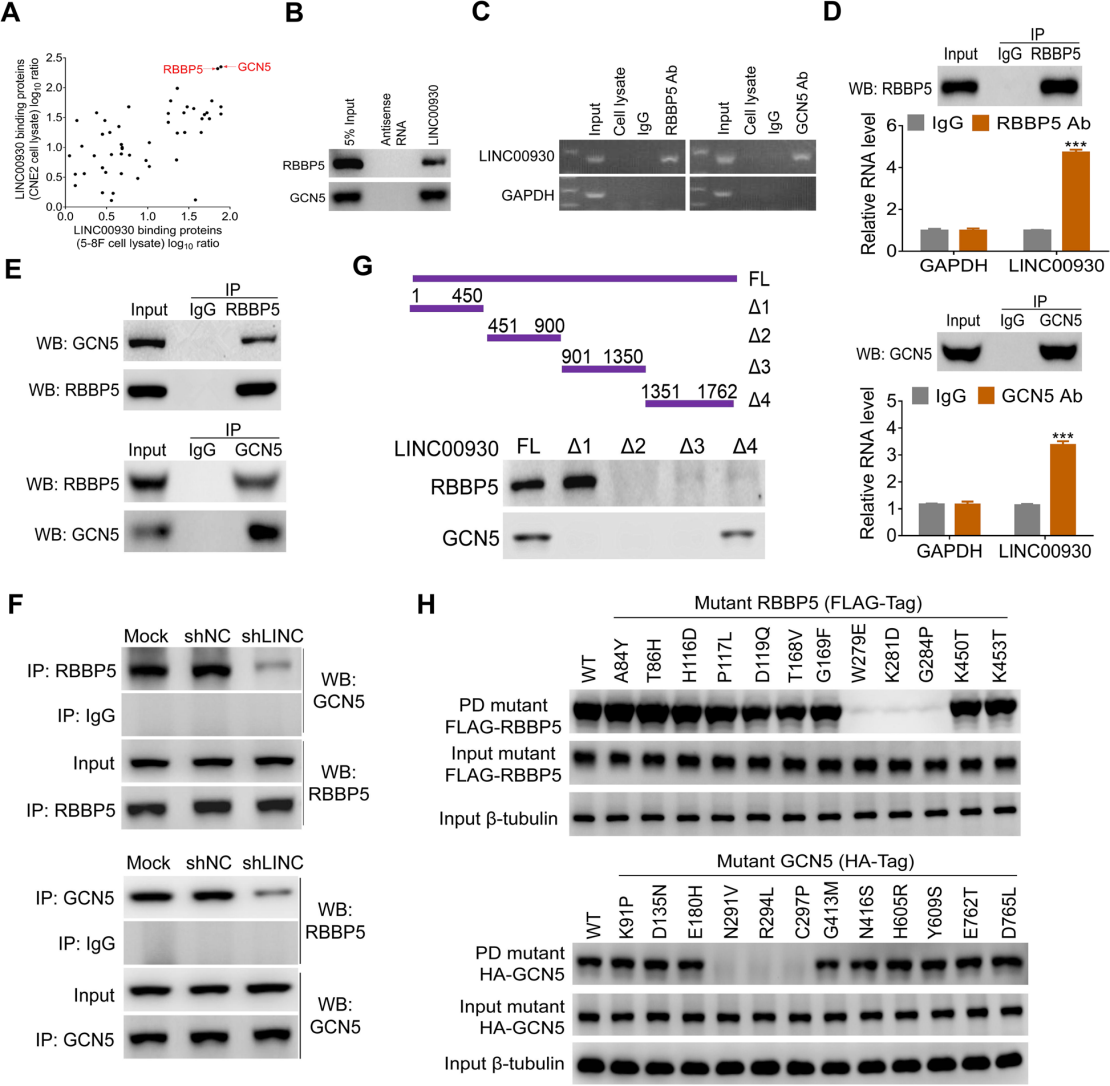
LINC00930 interacts with RBBP5 and GCN5. **a** The LINC00930-interacting proteins were annotated with their Log_10_ ratio in 5-8F and CNE2 cell lysate. **b** Western blot of the proteins from antisense LINC00930 and LINC00930 pull-down assays. **c** RNA immunoprecipitation experiments were performed using anti-RBBP5 or anti-GCN5 antibody, and specific primers were used to detect LINC00930 or GAPDH. **d** Nuclear lysates of 5-8F were immunoprecipitated with anti-RBBP5 antibodies (Left), anti-GCN5 antibody (Right), or control IgG. Aliquots of nuclear lysates (20% of input) and the RBBP5 or GCN5 immunoprecipitates were separated by SDS-PAGE, and the specific immunoprecipitation of RBBP5 and GCN5 was confirmed by western blot (WB). The complexes were analyzed for the presence of LINC00930 or GAPDH by real-time PCR. **e** Co-IP assay detected the interaction of RBBP5 and GCN5 in the 5-8F cell. The 20% of input (cell lysate) and RBBP5 or GCN5 immunoprecipitates were separated by SDS-PAGE. The specific immunoprecipitation of RBBP5 and GCN5 was confirmed by Western blot. **f** IP assay was performed to detect the interaction between RBBP5 and GCN5 after LINC00930 knockdown. **g** Western blot of RBBP5 and GCN5 in samples pulled down by full-length (FL) or truncated LINC00930 (Δ1: 1–450, Δ2: 451–900, Δ3: 901–1350, Δ4: 1351–1762). **h** Top: Mutation on W279, K281 and G284 of RBBP5 impaired the RBBP5-LINC00930 association. Bottom: Mutation on N291, R294 and C297 of GCN5 impaired the GCN5-LINC00930 association

Next, we set to elucidate the molecular mechanisms by which LINC00930 regulates PFKFB3. We first analyzed the location of LINC00930 in NPC cells. QRT-PCR (Fig. S4a) and ISH based on NPC cell lines (Fig. S4b) and tissues (Fig. S1e) consistently showed that LINC00930 was mainly located in the nucleus. Nuclear-localized lncRNAs usually function by physically interacting with transcriptional factors and histone regulators. Thus, we investigated whether LINC00930 interacts with certain cellular proteins to regulate gene expression. Using RNA pull-down assays, we identified a LINC00930-protein complex. The antisense strand of LINC00930 was used as a negative control. The mass spectrometry analysis results showed that LINC00930 bound numerous proteins in 5-8F and CNE2 cells (Supplementary Table S6 and Table S7). Gene ontology term enrichment (GO) analysis revealed that LINC00930-interacting proteins were associated with regulation of histone modification, cell proliferation, transcription initiation, and cell cycle (Fig. S4c). RBBP5 and GCN5 were selected for further validation because of their strong interactions with LINC00930 in both cells (Fig. 4a). Then, the RNA pull-down assay confirmed that LINC00930 physically interacted with RBBP5 and GCN5 in NPC cells (Fig. 4b).

RBBP5 is a core member of MLL/SET (mixed lineage leukemia/set-domain containing) histone-methyltransferase complexes and promotes histone H3 Lys 4 (H3K4) trimethylation and subsequent transcriptional activation [33, 34]. GCN5 functions as histone acetyltransferase (HAT) that enhances histone H3 Lys9 (H3K9) acetylation levels in the gene promoter [35, 36]. We thus postulated that LINC00930 served as a scaffold and mediated the interaction of RBBP5 and GCN5. This notion was supported by the following pieces of evidence: 1) RIP assay consolidated the interaction of LINC00930 and RBBP5 and GCN5 (Fig. 4c); 2) significantly increased enrichments of LINC00930 were observed in the anti-RBBP5 (4.8-fold) and anti-GCN5 (3.6-fold) immunoprecipitates compared with the IgG control (Fig. 4d); 3) IP analyses showed that RBBP5 and GCN5 physically interacted with each other (Fig. 4e); 4) LINC00930 knockdown did not affect RBBP5 and GCN5 expression (Fig. S4d); 5) LINC00930 knockdown greatly reduced the interaction between RBBP5 and GCN5 (Fig. 4f and Fig. S4e).

To determine which specific region within LINC00930 contributing to the binding with RBBP5 and GCN5, we constructed and biotinylated four fragments of LINC00930 (1–450 Δ1, 451–900 Δ2, 901–1350 Δ3, 1351–1762 Δ4) and used them in the pull-down assay with 5-8F cell lysates. We found that the 5′ fragment and 3′ fragment of LINC00930 mediated the interaction with RBBP5 and GCN5, respectively (Fig. 4g). To identify the LINC00930-interacting regions of RBBP5 and GCN5, we constructed 12 single amino acids mutations following catRAPID computational predictions (Fig. S4f). RIP results demonstrated that mutants of RBBP5 at W297, K281 and G284 completely attenuated the interaction between RBBP5 and LINC00930 (Fig. 4h, top); while mutants of GCN5 at N291, R294 and C297 thoroughly attenuated the interaction between GCN5 and LINC00930 (Fig. 4h, bottom). Taken together, LINC00930 interacts with RBBP5 and GCN5 epigenetic modification complex.

### LINC00930 is a molecular link between RBBP5/GCN5 and PFKFB3

**Fig. 5:**
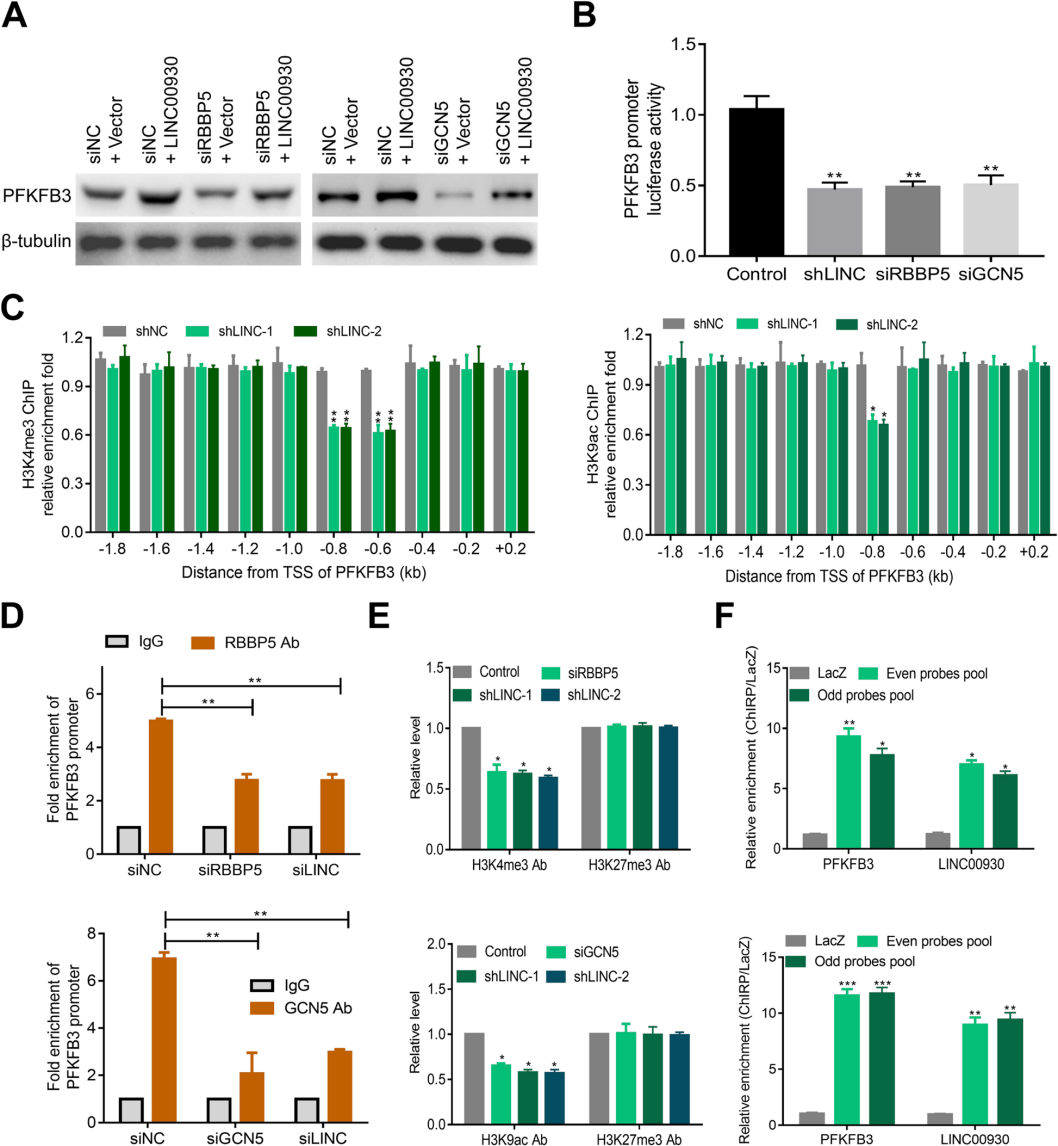
LINC00930 functions as a link between RBBP5/GCN5 and PFKFB3. **a** Western blot assay was performed to measure PFKFB3 level after transfection of LINC00930-overexpressing plasmid, RBBP5 or GCN5 siRNAs in CNE1 cells. **b** Luciferase reporter vector was generated by inserting the promoter region (− 2000 bp to + 200 bp) of the PFKFB3 gene. The reporter vectors were then cotransfected into CNE2 cells with LINC00930 shRNA, RBBP5 or GCN5 siRNAs. Cells were harvested for luciferase activity assay. **c** ChIP-qPCR was performed to evaluate the enrichment of H3K4me3 or H3K9ac in different promoter regions of PFKFB3 in CNE2 cells after knocking down LINC00930. **d** Real-time PCR of the ChIP samples was applied to detect the binding efficiency of RBBP5 or GCN5 to the PFKFB3 gene promoter (− 800 bp to − 600 bp) after transfection of RBBP5, GCN5, or LINC00930 siRNAs in CNE2 cells, respectively. **e** Real-time PCR of the ChIP samples was utilized to measure the H3K4 trimethylation or H3K9 acetylation and H3K27 trimethylation levels of the PFKFB3 gene promoter (− 800 bp to − 600 bp) after knockdown of RBBP5, GCN5, or LINC00930 in CNE2 cells, respectively. **f** ChIRP assays of the enrichment of LINC00930 and PFKFB3 promoter (− 800 bp to − 600 bp) in both even and odd probes pools relative to control LacZ probes set in 5-8F and CNE2 cells. Data in **b, c, d**, **e** & **f** are presented as mean ± SEM of three independent experiments. The *p*-value was determined by a two-tailed unpaired Student’s *t* test. * *p* < 0.05; ** *p* < 0.01

Given that we showed that LINC00930 interacted with RBBP5 and GCN5, we postulated that GCN5-mediated histone acetylation and RBBP5-mediated histone methylation are involved in PFKFB3 transcription. Several pieces of evidence confirm our hypothesis. First, PFKFB3 was upregulated upon Trichostatin A (a pan-inhibitor of class I and II histone deacetylases [37]) treatment. Second, YF-2 (an inhibitor of GCN5 network) treatment, but not curcumin (an inhibitor of EP300/CREBBP) or anacardic acid (AA, an inhibitor of p300/CBP), reduced the luciferase activity of PFKFB3 in NPC cells (Fig. S5b), suggesting that the transcriptional activity of PFKFB3 was regulated by GCN5-mediated histone acetylation modification. Third, treatment with OICR-9429, a small-molecule antagonist of the MLL/SET domain histone-methyltransferase complexes [38], significantly inhibited the expression (Fig. S5c) and transcriptional activity of PFKFB3 (Fig. S5d). In addition, RT-PCR and western blot results showed that RBBP5 or GCN5 knockdown remarkably weakened LINC00930-induced PFKFB3 upregulation (Fig. 5a, Fig. S5e-g). In the end, RBBP5, GCN5 or LINC00930 silencing obviously inhibited the luciferase activity of PFKFB3 (Fig. 5b). Collectively, these data indicate that LINC00930 transactivates PFKFB3 via RBBP5 and GCN5 mediated epigenetic modification.

Then, 10 pairs of primers (Supplementary Table S4) were used to detect potential interacting sites in PFKFB3 promoter region. As shown in Fig. 5c, ChIP-qPCR assay indicated that the enrichments of H3K4me3 and H3K9ac were both obviously decreased at mostly from − 800 bp to − 600 bp promoter region of PFKFB3 after LINC00930 knockdown. Further ChIP-qPCR analyses indicated that knockdown of RBBP5 or GCN5 significantly decreased the binding efficiency of RBBP5 or GCN5, respectively (Fig. 5e); while knockdown of LINC00930 decreased the binding efficiencies of both RBBP5 and GCN5 (Fig. 5e). Consistently, ChIRP analyses confirmed the direct interaction between LINC00930 and PFKFB3 promoter region (Fig. 5f). In the end, LINC00930 overexpression failed to activate transcription when ~ − 1, 000 bp to ~ − 500 bp region of promoter of PFKFB3 was deleted (Fig. S5h). Taken together, our data suggest that LINC00930 acts as a modular scaffold of RBBP5 and GCN5 complexes, which results in the enrichment of H3K4me3 and H3K9ac in the promoter regions of PFKFB3, and activates its transcription.

### LINC00930 promotes NPC progression via PFKFB3

**Fig. 6:**
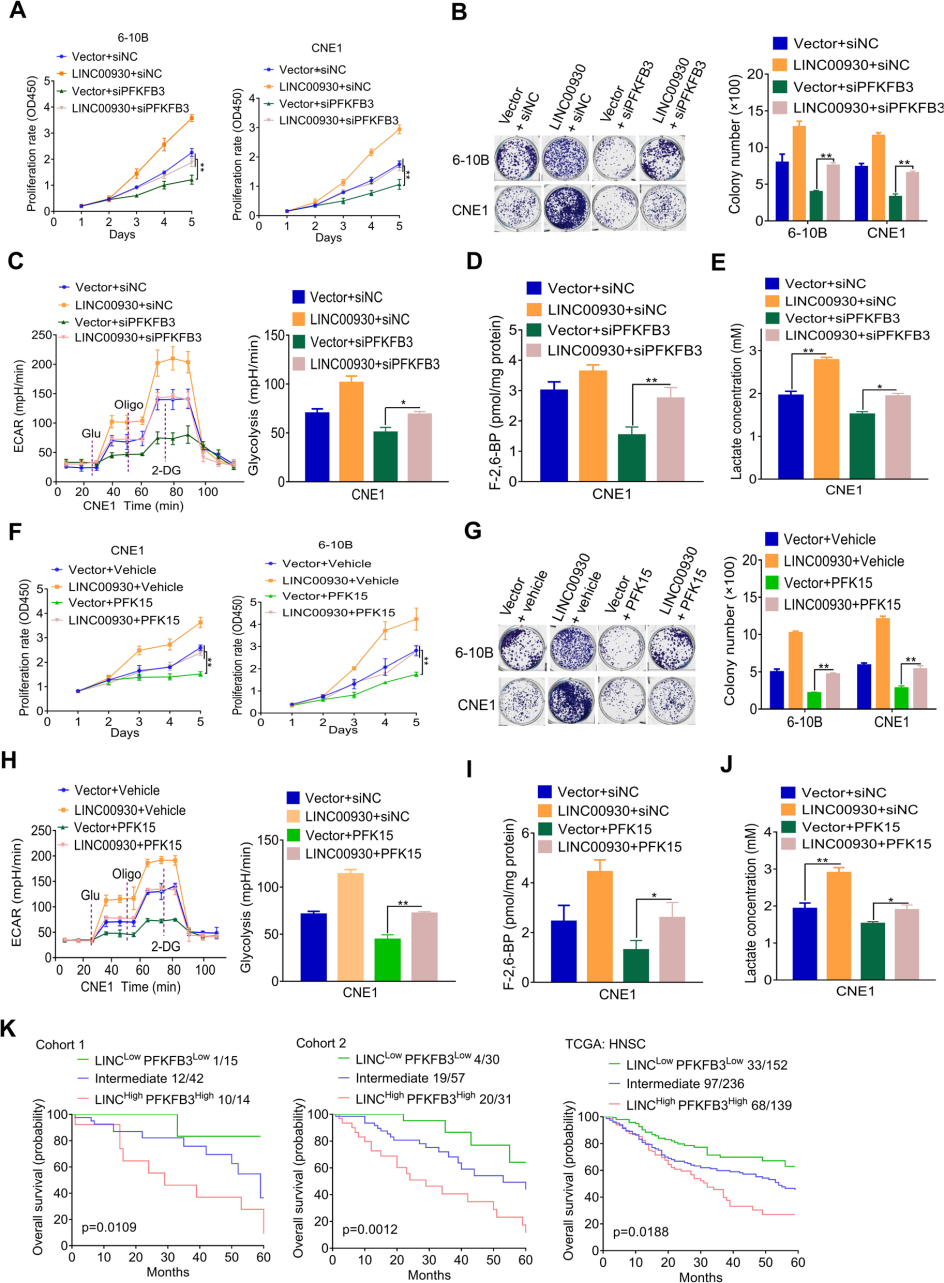
LINC00930 promotes NPC progression via PFKFB3. **a** and **b** Cell viability and colony formation was assessed after transfection of the PFKFB3 siRNA in LINC00930-overexpressing 6-10B and CNE1 cells. **c** The ECAR was measured and analyzed after transfection of the the PFKFB3 siRNA in LINC00930-expressing CNE1 cells. **d** and **e** Intracellular F-2,6-BP level and lactate level were assayed after transfection of the PFKFB3 siRNA in LINC00930-expressing CNE1 cells. **f** and **g** Cell viability and colony formation was assessed after treatment with PFKFB3 inhibitor PFK15 in LINC00930-overexpressing 6-10B and CNE1 cells. **h** The ECAR was measured and analyzed after treatment with PFKFB3 inhibitor PFK15 in LINC00930-overexpressing CNE1 cells. **i** and **j** Intracellular F-2,6-BP level and lactate level were detected after treatment with PFKFB3 inhibitor PFK15 in LINC00930-overexpressing CNE1 cells. **k** Kaplan-Meier analysis of overall survival of patients in cohort 1, cohort 2, and TCGA HNSC cohort with low (low expression of both LINC00930 and PFKFB3), high (high expression of both LINC00930 and PFKFB3) or intermediate LINC00930/PFKFB3 expression (log-rank test, two-sided). The *p*-value in **a & f** was determined by one-way analysis of variance (ANOVA) with Dunnett’s multiple comparisons test, no adjustments were made for multiple comparisons. The *p*-value in **b, c, d, e, g, h, i** & **j** was determined by a two-tailed unpaired Student’s *t* test. * *p* < 0.05; ** *p* < 0.01; *** *p* < 0.001

Next, we would like to investigate whether PFKFB3 modulates the biological function of LINC00930 in NPC. First, LINC00930 overexpression significantly promoted the cell proliferation (Fig. 6a) and colony formation (Fig. 6b) of NPC cells; while PFKFB3 knockdown substantially attenuated the oncogenic effect induced by the LINC00930 (Fig. 6a and b). Consistently, PFKFB3 silencing significantly blocked the glycolytic activity (Fig. 6c) and reduced F-2,6-BP (Fig. 6d) and lactate levels (Fig. 6e) induced by LINC00930. PFK15 has been reported to be a specific and potent small molecule antagonist of PFKFB3, and is able to suppress the proliferation of various cancer cells^9^. Consistently, treatment with PFK15 for 24 h indeed weakened the oncogenic effect induced by the LINC00930, including cell proliferation (Fig. 6f and g), tumor glycolysis (Fig. 6h), F-2,6-BP (Fig. 6i) and lactate levels (Fig. 6j). Together, PFKFB3 mediates the oncogenic function of LINC00930 in NPC cells.

In light of the above findings, we investigated the clinical significance of LINC00930 and PFKFB3 in NPC patients. Patients from cohort 1, cohort 2 and the TCGA HNSC cohort were sorted into the LINC00930/PFKFB3-high, LINC00930/PFKFB3-intermediate, and LINC00930/PFKFB3-low groups, respectively. Subsequently, we calculated OS for NPC patients by means of the combined index of LINC00930 and PFKFB3 expression. As shown in Fig. 6k, NPC patients with LINC00930/PFKFB3-high subset tended to have the worst prognosis compared to the other two subsets. These data further confirm that LINC00930 and PFKFB3 both have oncogenic function. Targeting LINC00930 and PFKFB3 simultaneously may be a promising approach to treat NPC patients.

### Targeting LINC00930 and PFKFB3 in combination with radiotherapy induces tumor regression

**Fig. 7:**
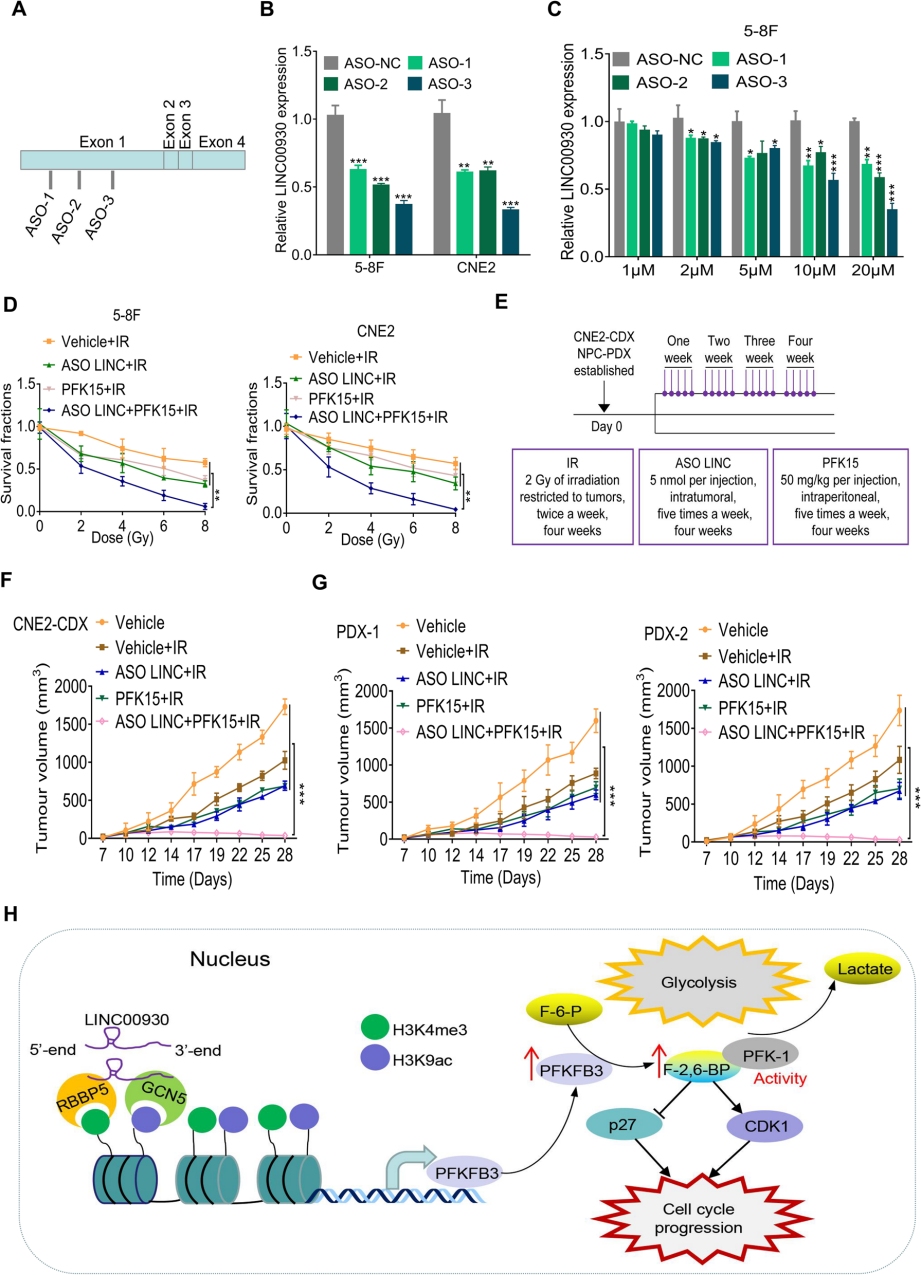
Targeting LINC00930 and PFKFB3 in combination with radiotherapy induces tumor regression. **a** Schematic view of ASO targets on LINC0930 mRNA. **b** Relative expression of LINC00930 48 h after ASO transfection were determined by qRT-PCR. **c** qRT-PCR showed LINC00930 expression after ASOs delivery without transfect reagents. ASO were added into 5–8 cells at the concentration as indicated. After 48 h, RNA was extracted, and qRT-PCR was performed. **d** Cell viability was measured by CCK-8 assay afer irradiation of NPC cells treated with ASO LIN00930 or (and) PFK15. IR, Irradiation. **e** Schematic view of IR, ASO LINC00930 or (and) PFK15 treatment in CNE2-CDX and NPC-PDX xenograft model. CDX, cell-derived xenograft; PDX, patient-derived xenograft. **f** and **g** Statistical analysis of tumor volume in the CNE2-CDX, PDX-1, and PDX-2 models. IR, Irradiation. **h** Graphical abstract showing that the lncRNA LINC00930 promotes tumor glycolysis and cell proliferation by epigenetically upregulating PFKFB3. The *p*-value in **b & c** was determined by a two-tailed unpaired Student’s *t* test. The *p*-value in **d, f** & **g** was determined by one-way analysis of variance (ANOVA) with Dunnett’s multiple comparisons test, no adjustments were made for multiple comparisons. * *p* < 0.05; ** *p* < 0.01; *** *p* < 0.001

Antisense oligonucleotides (ASOs) are short, chemically synthesized single-stranded oligonucleotides with good stability, target binding properties, and low biological toxicity [39, 40]. Here, we designed and synthesized three ASOs of LINC00930. Results showed that all three ASOs successfully knocked down LINC00930 (Fig. 7a and b), and the knockdown efficiency of ASO was concentration-dependent (Fig. 7C and Fig. S6a).

Combination of radiotherapy and chemotherapy is an important treatment strategy for NPC. We hypothesized that LINC00930 and PFKFB3 depletion in combination with radiotherapy might have a better anti-tumor effect. First, we found that ASO LINC00930 and PFK15 treatment sensitized 5-8F and CNE2 cells to irradiation (Fig. 7d). Then, orthotopic xenograft tumor model was used to determine the therapeutic efficacy. CNE2 cell-based xenograft and two NPC patient-derived xenograft (PDX) models were established and therapy method was present in Fig. 7e. Tumor growth curve showed that either LINC00930 depletion via in vivo-optimized LINC00930 inhibitor (ASO LINC) or PFKFB3 inhibitor (PFK15) alone could enhance radiosensitivity of tumors, while the combination of both inhibitors treatment and radiotherapy induced tumor regression of a CNE2 cell-derived xenograft (CDX) and two NPC patients-derived xenografts (PDX) (Fig. 7f, g and S6b). It is worth noting the drug combination was well-tolerated as the body weights of mice were maintained during the course of the drug treatments (Fig. S6c). Accordingly, as shown in Fig. S6d and S6e, LINC00930 and PFKFB3 inhibitors significantly reduced F-2,6-BP and lactate levels in CDX tumors. Considering the side effects, we evaluated the potential effects of ASO LINC and PFK15 on major organs. Histopathological changes in the liver and kidney were analyzed. No significant morphological changes were observed in these organs (Fig. S5f). In addition, we found that single or dual drugs in combination with radiotherapy had slight effects on the levels of ALT and Cr in the serum of mice (Fig. S5g and S5h). The above results showed that treatment with ASO LINC or PFK15 not only achieved a significant anti-tumor effect but also had no obvious adverse effects. Together, our data suggest that targeting LINC00930 and PFKFB3 could be an effective approach to enhance radiosensitivity of NPC patients.

## Discussion

Metabolic reprogramming is one of the key characteristics of malignant tumor. Recently, a flow of researches have implicated that lncRNAs were involved in reprogramming energy metabolism and regulated cell proliferation and tumor progression [5, 12, 14, 17, 41, 42]. However, how lncRNA regulates cellular energy metabolism, especially glucose metabolism in NPC, remains largely unknown. Here, we report a novel metabolism-related and clinically relevant lncRNA, LINC00930, which significantly influences tumor glycolysis and cell proliferation by modulating the interaction with the RBBP5 and GCN5 epigenetic remodeling complex to further alter the pattern of histone modification and transactivating the target gene PFKFB3 in NPC.

The Warburg effect, which represents a shift in the way tumor cells utilize glucose from oxidative phosphorylation to glycolysis, is now considered a major feature of tumors. This change in energy metabolism is regulated by complex factors [43, 44]. When we explored the mechanisms by which LINC00930 regulates glycolysis, we found the involvement of PFKFB3. One of the critical role of PFKFB3 is to catalyze the conversion of F-6-P to F-2,6-BP, thus activating glycolysis and promoting tumor progression [11, 31]. Apart from the well-documented role in glycolysis activation, recent observations have established roles of PFKFB3 beyond glycolysis. The product of PFKFB3, F-2,6-BP increases cell cycle inhibitor p27 phosphorylation at Thr-187, which activating p27 ubiquitination and degradation, and thus promoting G1/S transition [10, 11]. In this study, we demonstrated that LINC00930 epigenetically upregulated PFKFB3 and activating glycolysis process and cell cycle progression at the G1/S phase transition, thus regulating NPC cell proliferation and tumor growth. The data consistently suggest that LINC00930 is an tumor-promotive lncRNA in NPC.

LncRNAs are involved in the regulation of gene expression at the transcriptional and post-transcriptional levels. First, lncRNAs regulate transcriptional expression by blocking promoter regions, interacting with RNA-binding proteins, or modulating the activity of transcription factors. Second, lncRNAs recruit a chromatin remodeling complex to specific sites and regulate expression processes [5, 21, 45]. In this study, we demonstrated that LINC00930 was mainly located in the nucleus and directly interacted with chromatin remodeling proteins, RBBP5 and GCN5. Further, we established specific region within LINC00930 and key amino acid residues within RBBP5/GCN5 involved in this RNA-protein intact complex. We found that LINC00930 activated the transcription of downstream target gene PFKFB3 by regulating histone modification. This notion was verified by three lines of experimental evidence: (i) LINC00930 physically interacted with RBBP5 and GCN5; (ii) pharmacological inhibitors of histone methylation and acetylation reduced the luciferase activity of PFKFB3; (iii) LINC00930 knockdown decreased the H3K4me3 and H3K9ac levels at the promoter region of PFKFB3. Further, PFKFB3 was functionally responsible for LINC00930-mediated nasopharyngeal carcinogenesis. Collectively, our findings establish a novel mechanism of lncRNA regulating tumor metabolism, of which LINC00930 regulates the key glycolytic gene PFKFB3 by epigenetic modification.

Enhancing the understanding of the molecular mechanisms underlying NPC may promote the development of effective target therapy and improve the overall prognosis of patients with this disease [3, 46]. In recent years, novel molecular targeted therapy has achieved remarkable curative effects in clinical practice, which shows the correctness and feasibility of tumor targeted therapy theory, thus pushing NPC therapy to a new stage. Molecular targeted therapy in combination with radiotherapy appears to be a promising event for NPC comprehensive therapy strategy [47]. Our study reported that high expression of LINC00930 and PFKFB3 was significantly associated with a poor prognosis in NPC. Further, we demonstrated via the in vivo therapeutic models that treatment with LINC00930 inhibitor and PFKFB3 inhibitor in combination with radiotherapy induced tumor regression, suggesting that targeting LINC00930 and PFKFB3 could be an effective approach to enhance radiosensitivity of NPC patients. We are actively pursuing a clinical strategy to treat nasopharyngeal carcinoma by interfering with LINC00930 and the target gene PFKFB3.

## Conclusion

In conclusion, our study identifies LINC00930 as an oncogenic lncRNA, which promotes PFKFB3-mediated glycolysis vis histone modification. These findings imply that targeting LINC00930 and PFKFB3 could be an effective approach to enhance radiosensitivity of NPC patients.

## Supplementary Information

**Additional file 1: Fig. S1.** Related to Fig. 1 LINC00930 is a metabolism-related lncRNA and clinically relevant with the progression of NPC. **Fig. S2.** Related to Fig. 2 LINC00930 promotes cell proliferation and glycolysis. **Fig. S3.** Related to Fig. 3 LINC00930 correlates with and regulates PFKFB3. **Fig. S4.** Related to Fig. 4 LINC00930 interacts with RBBP5 and GCN5. **Fig. S5.** Related to Fig. 5. LINC00930 functions as a link between RBBP5/GCN5 and PFKFB3. **Fig. S6.** Related to Figs. 6 and 7 Potential therapeutic role of LINC00930 in NPC.

**Additional file 2: Supplementary Table S1.** Clinicopathological data of 71 cases primary NPC biopsies in cohort 1. **Supplementary Table S2.** Clinicopathological data of 128 cases paraffin-embedded NPC biopsies in cohort 2. **Supplementary Table S3.** Details of shRNAs and siRNAs target sequences. **Supplementary Table S4.** Primers used in the study. **Supplementary Table S5.** Differentially regulated lncRNAs in NPC. **Supplementary Table S6.** Proteins interacted with LINC00930 identified by Liquid Chromatography-Mass Spectrometry in 5-8F cell. **Supplementary Table S7.** Proteins interacted with LINC00930 identified by Liquid Chromatography-Mass Spectrometry in CNE2 cell.

## Data Availability

Datasets used and/or analyzed during the current study are available from the corresponding author on reasonable request.

## Abbreviations

LncRNA: Long noncoding RNA
ISH: In situ hybridization
TCGA: The cancer genome atlas
IP: Immunoprecipitation
RIP: RNA immunoprecipitation
Co-IP: Coimmunoprecipitation
ChIP: Chromatin immunoprecipitation
CDX: Cell-derived xenograft
PDX: Patient-derived xenograft
NPC: Nasopharyngeal carcinoma
DYS: Dysplasia
GEO: Gene Expression Omnibus
PD: Pathological differentiation
PFKFB3: Phosphofructo-2-kinase/fructose-2,6-biphosphatase 3
FDR: False discovery ratio
IHC: Immunohistochemistry
NPE: Nasopharyngeal epithelium
SM: Squamous metaplasia
ECAR: Extracellular acidification rate
HAT: Histone acetyltransferases
ASO: Antisense oligonucleotide
