# Supplementary material for: Long noncoding RNA LINC00930 promotes PFKFB3-mediated tumor glycolysis and cell proliferation in nasopharyngeal carcinoma
Source: J Exp Clin Cancer Res. 2022 Feb 24;41:77. doi: 10.1186/s13046-022-02282-9 (PMC8867671; doi:10.1186/s13046-022-02282-9)
Supplement: Supplementary file 1 — Additional file 1: Fig. S1. Related to Fig. 1 LINC00930 is a metabolism-related lncRNA and clinically relevant with the progression of NPC. Fig. S2. Related to Fig. 2 LINC00930 promotes cell proliferation and glycolysis. Fig. S3. Related to Fig. 3 LINC00930 correlates with and regulates PFKFB3. Fig. S4. Related to Fig. 4 LINC00930 interacts with RBBP5 and GCN5. Fig. S5. Related to Fig. 5. LINC00930 functions as a link between RBBP5/GCN5 and PFKFB3. Fig. S6. Related to Figs. 6 and 7 Potential therapeutic role of LINC00930 in NPC. [file 13046_2022_2282_MOESM1_ESM.pdf]

Additional file 1
Supplementary Figures and Legends

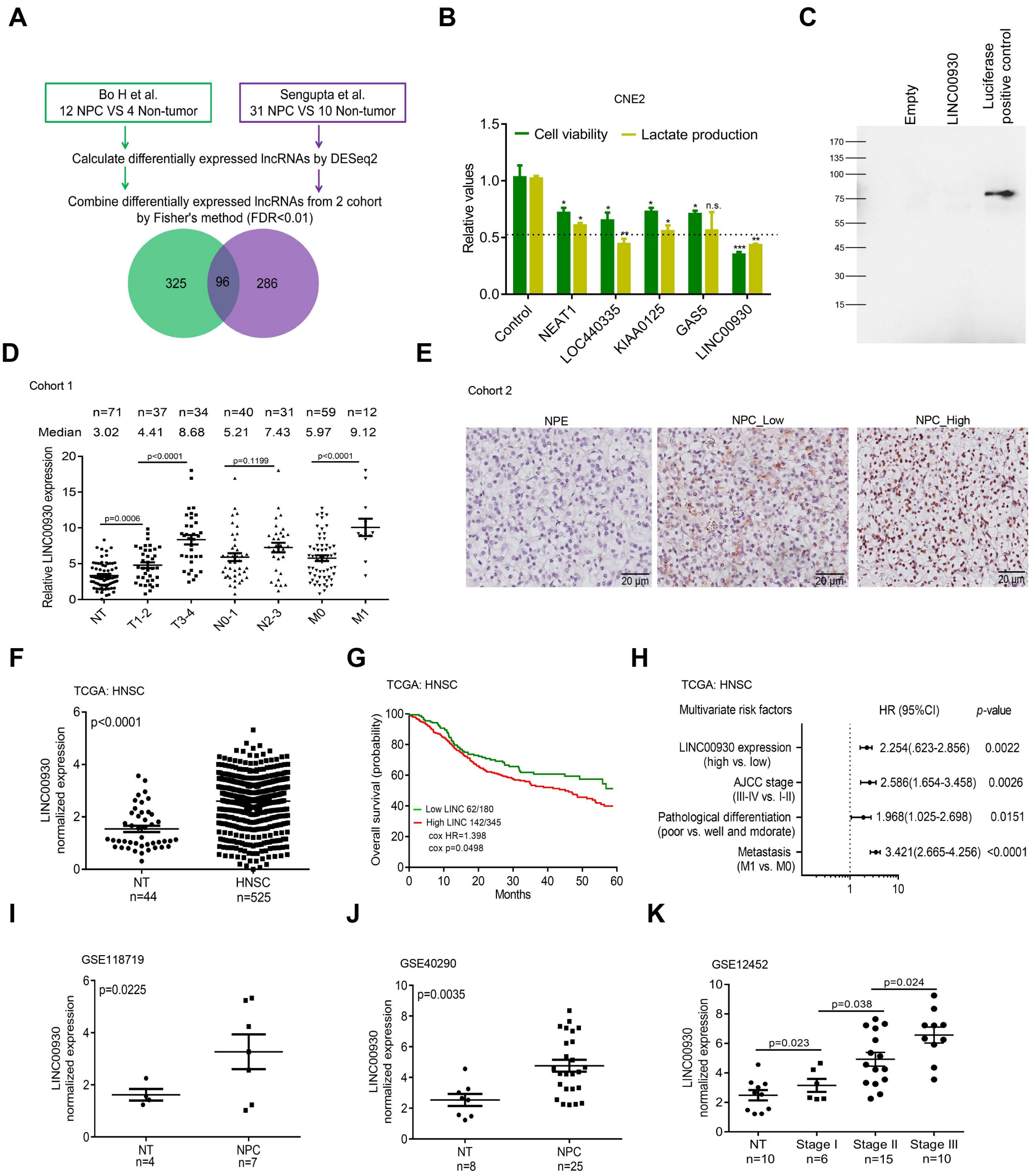

Figure S1 related to Fig. 1 LINC00930 is a metabolism-related lncRNA and clinically relevant with the progression of NPC. **a** Workflow showing the steps in the discovery and identification of clinically relevant lncRNAs in two well-established NPC cohorts. **b** Five lncRNAs regulated both cell proliferation and lactate production in CNE2 cells, n = 3 biologically independent samples. **c** In translation assay, a 4966-bp genomic region containing LINC00930 was cloned into a pcDNA vector and expressed using the TnT Quick Coupled Transcription/Translation System. The absence of a specific band indicated that LINC00930 is a transcript with no protein-coding capacity. Luciferase in vitro translation served as positive control. **d**

LINC00930 was measured in tissues from NPC patients of different TNM subgroups in cohort 1 (n = 71, nonparametric Mann-Whitney test). **e** Representative images of LINC00930 expression in NPC and adjacent nasopharyngeal epithelium using ISH analysis in cohort 2 (n = 128). **f** LINC00930 expression levels were analyzed in tumors compared to corresponding non-tumor tissues (NT). The RNA-seq data of tumors and matched non-tumor controls were downloaded from the TCGA website (Nonparametric Mann-Whitney test). HNSC, head and neck squamous cell carcinoma. **g** Survival was analyzed and compared between patients with high and low levels of LINC00930 expression in tumor in TCGA HNSC group (n = 525, log-rank test, two-sided). **h** Multivariable analysis was performed in TCGA HNSC cohort. All the bars correspond to 95% confidence intervals. **i-j** LINC00930 expression levels were analyzed in two cohort of NPC samples from the GEO database repository (GSE118719 and GSE40290, nonparametric Mann-Whitney test). **k** LINC00930 expression was associated with clinical AJCC stages in GSE12452 dataset (n = 31, nonparametric Mann-Whitney test).

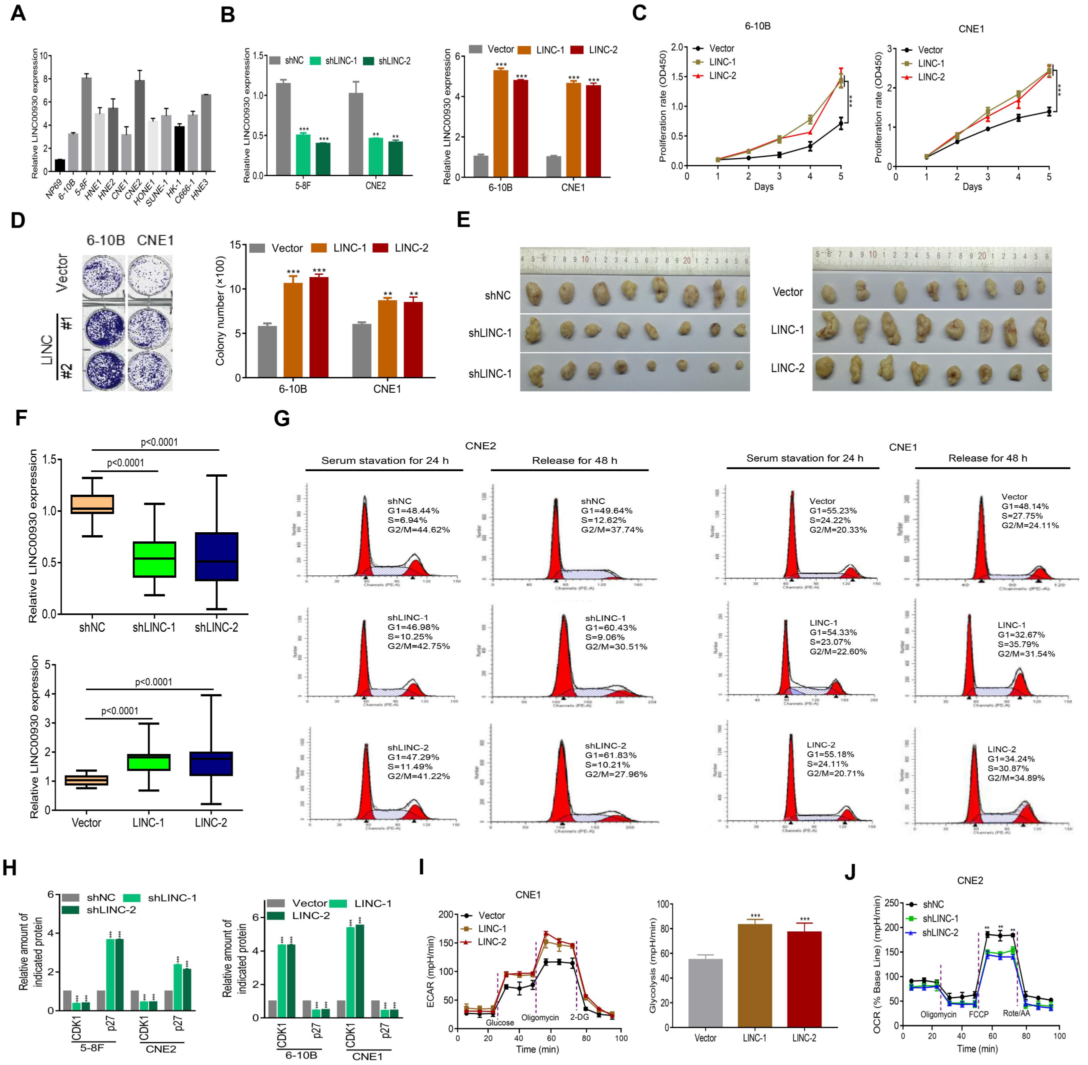

Figure S2 related to Fig. 2 LINC00930 promotes cell proliferation and glycolysis. **a** The mRNA level of LINC00930 in immortalized normal nasopharyngeal epithelial NP69 cell and human NPC cell lines was determined by real-time PCR. **b** 5-8F, CNE2 cells with silencing LINC00930 expression and 6-10B, CNE1 cells with LINC00930 overexpression were stably established by lentivirus transduction. **c** CCK-8 assays of 6-10B and CNE1 cells stably overexpressing LINC00930. **d** Colony formation assays of 6-10B and CNE1 cells stably overexpressing LINC00930. **e** Images of xenograft tumors derived from LINC00930-knockdown cells or LINC00930-overexpressing cells. **f** LINC00930 levels were detected by real-time PCR in xenograft tissues derived from CNE2 cells with LINC00930 knockdown and CNE1 cells with LINC00930 overexpression. Three different sites were selected from each xenograft tumor (n = 8). **g** The cell cycle distributions of CNE2 cell with LINC00930

knockdown or CNE1 cell with LINC00930 overexpression were examined by flow cytometry analysis. **h** Quantification of Western blots shown in Fig. 2f by Image J. **i** The ECAR was measured in CNE1 cells with LINC00930 overexpression using an XF Extracellular Flux Analyzer. **j** OCR was detected in CNE2 cells with LINC00930 knockdown. The p-value in **a, b, d, e, f, g & h** was determined by a two-tailed unpaired Student's *t* test. The p-value in **c** was determined by one-way analysis of variance (ANOVA) with Dunnett's multiple comparisons test, no adjustments were made for multiple comparisons. * $p < 0.05$; ** $p < 0.01$; *** $p < 0.001$.

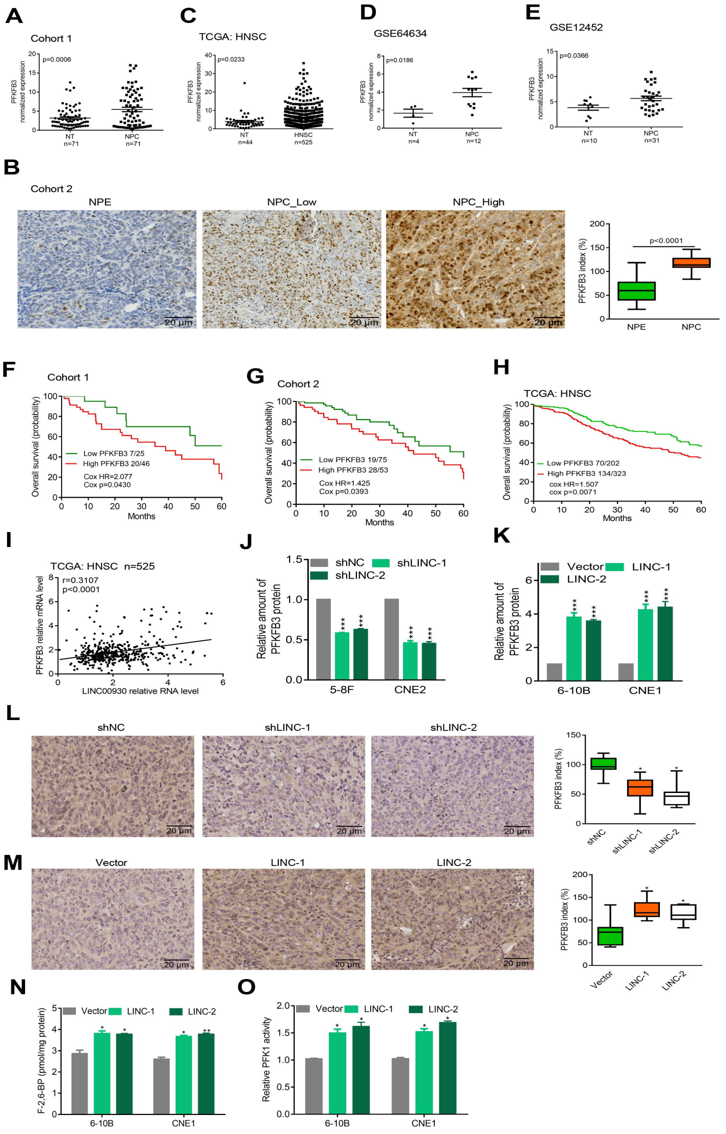

Figure S3 related to Fig. 3 LINC00930 correlates with and regulates PFKFB3. **a** Analysis of PFKFB3 expression levels in NPC tissues and normal tissues in cohort 1 (n = 71, non-parametric Mann-Whitney test). **b** Representative images of PFKFB3 expression in NPC and adjacent nasopharyngeal epithelium using IHC analysis in cohort 2. **c** PFKFB3 expression level was analyzed in tumors and corresponding non-tumor tissue (NT) from TCGA HNSC dataset (n = 525, non-parametric Mann-Whitney test). **d** and **e** Upregulated LINC00930 expression was confirmed in NPC biopsies compared with non-tumor tissues in GSE64634 and GSE12452 dataset (Non-parametric Mann-Whitney test). **f-h** Survival was analyzed and compared between patients with low and high levels of PFKFB3 in cohort 1, cohort 2 and TCGA HNSC cohort (Log-rank test, two-sided). HR, Hazard Ratio. **i** The correlation

between LINC00930 transcript level and PFKFB3 mRNA level was analyzed using TCGA HNSC dataset (n = 525, spearman rank-correlation analysis). **j** and **k** Quantification of Western blots shown in Fig 3c and 3d by Image J. **l** and **m** PFKFB3 levels were detected using IHC analysis in xenograft tissues derived from LINC00930-knockdown cells or LINC00930-overexpressing cells. **n** and **o** F-2,6-BP level and PFK1 activity were detected in LINC00930-overexpressing 6-10B and CNE1 cells.

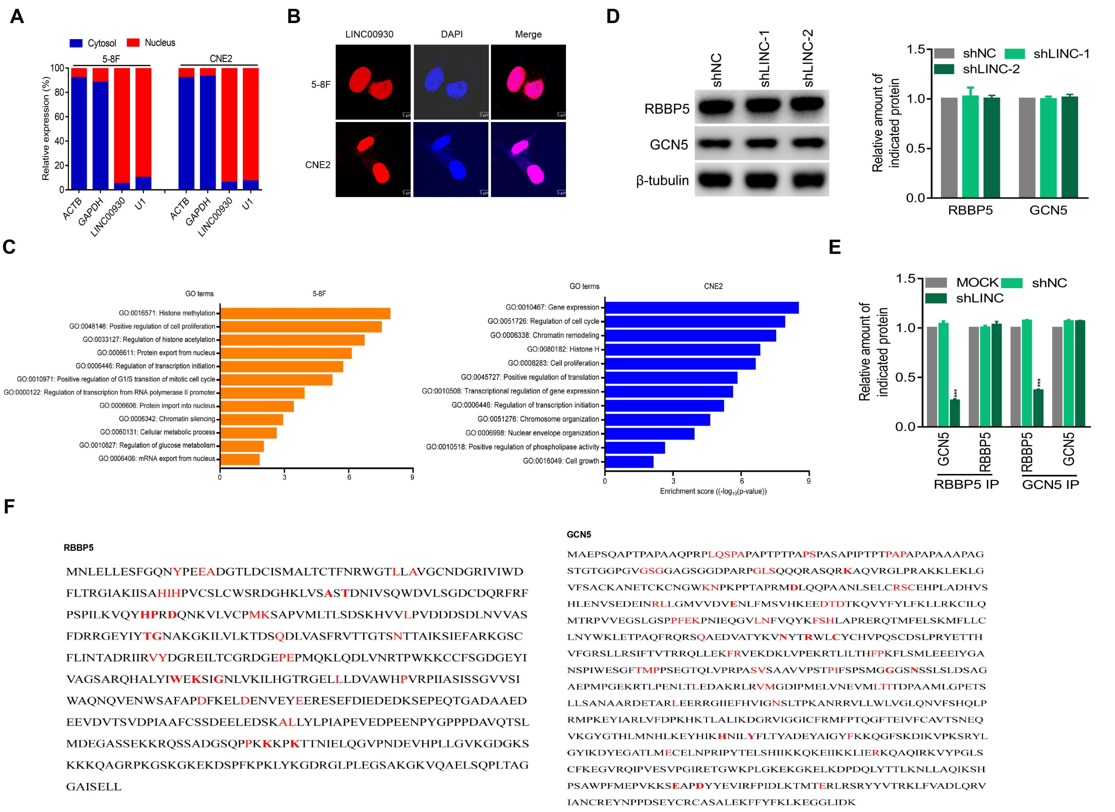

Figure S4 related to Fig. 4 LINC00930 interacts with RBBP5 and GCN5. **a** Expression of LINC00930 in cytoplasmic and nuclear fractionations of two NPC cell lines. ACTB and GAPDH serve as positive control for cytoplasmic gene expression. U1 RNA serves as a positive control for nuclear gene expression. **b** LINC00930 location was detected *in situ* by the RNA FISH assay in two NPC cell lines. LNA probe for LINC00930 was hybridized with fixed cells. DAPI was used to visualize nuclei. LINC00930 (red) and nuclei (blue) were visualized by a laser-scanning confocal microscope. **c** Gene ontology (GO) enrichment for LINC00930-interacting proteins in 5-8F and CNE2 cell lysate. **d** Western blot was performed to detect RBBP5 and GCN5 expression after transfection of LINC00930 shRNAs in 5-8F cells. **e** Quantification of Western blots shown in Fig. 4f by Image J. **f** Potential LINC00930-binding sites of RBBP5 and GCN5 are shown in red.

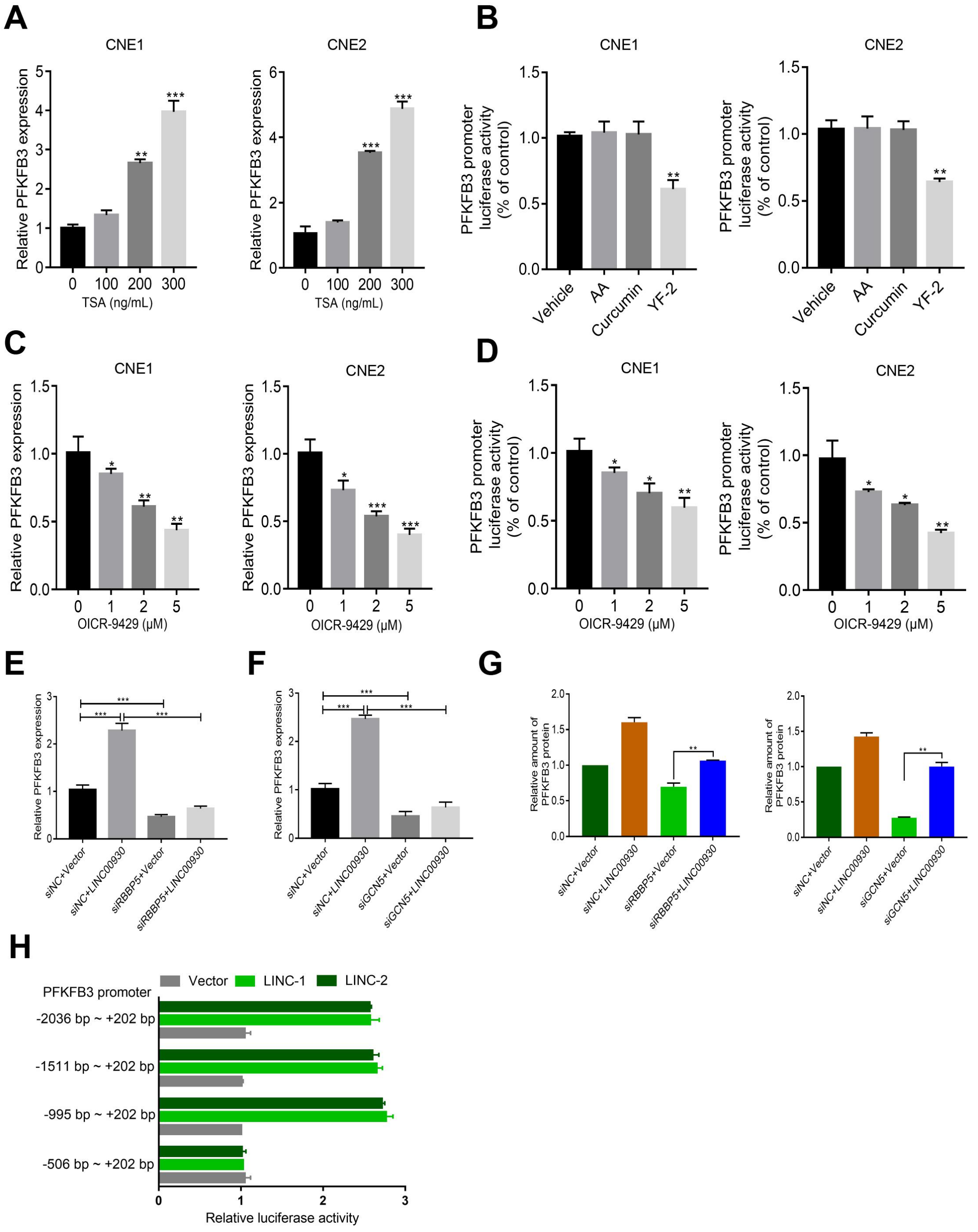

Figure S5 related to Fig. 5 LINC00930 functions as a link between RBBP5/GCN5 and PFKFB3. **a** PFKFB3 expression was measured by real-time PCR in CNE1 and CNE2 cells after the treatment of Trichostatin A for 24 h. **b** Cells were transfected with the PFKFB3-promoter 2kb-pGL3 and Renilla plasmids; after 24 h, the cells were treated with the indicated HAT inhibitors. After 6 h of treatment, a dual luciferase assay was performed. AA, anacardic acid. **c** PFKFB3 expression was measured by real-time PCR in CNE1 and CNE2 cells after the treatment of

OICR-9429 for 24 h. **d** Cells were transfected with the PFKFB3-promoter 2kb-pGL3 and Renilla plasmids; after 24 h, the cells were treated with OICR-9429. After 12 h of treatment, a dual luciferase assay was performed. **e** and **f** Real-time PCR assay was performed to measure PFKFB3 level after transfection of LINC00930-overexpressing plasmid, RBBP5/GCN5 siRNA and control siRNA plus control plasmid in CNE1 cells. **g** Quantification of Western blots shown in Fig. 5a by Image J. **h** LINC00930 overexpression cannot activate the transcription of PFKFB3 when ~ -1000 bp to ~ -500 bp promoter region of PFKFB3 was deleted. Empty vector or LINC00930 expressing vectors were co-transfected with the indicated reporter vectors with different promoter regions of PFKFB3 in CNE1 cells. Luciferase activities were assayed 48 hours post-transfection. Data in **a**-**g** are presented as mean ± SEM of three independent experiments. The p-values were determined by a two-tailed unpaired Student's *t* test. * $p < 0.05$; ** $p < 0.01$; *** $p < 0.001$.

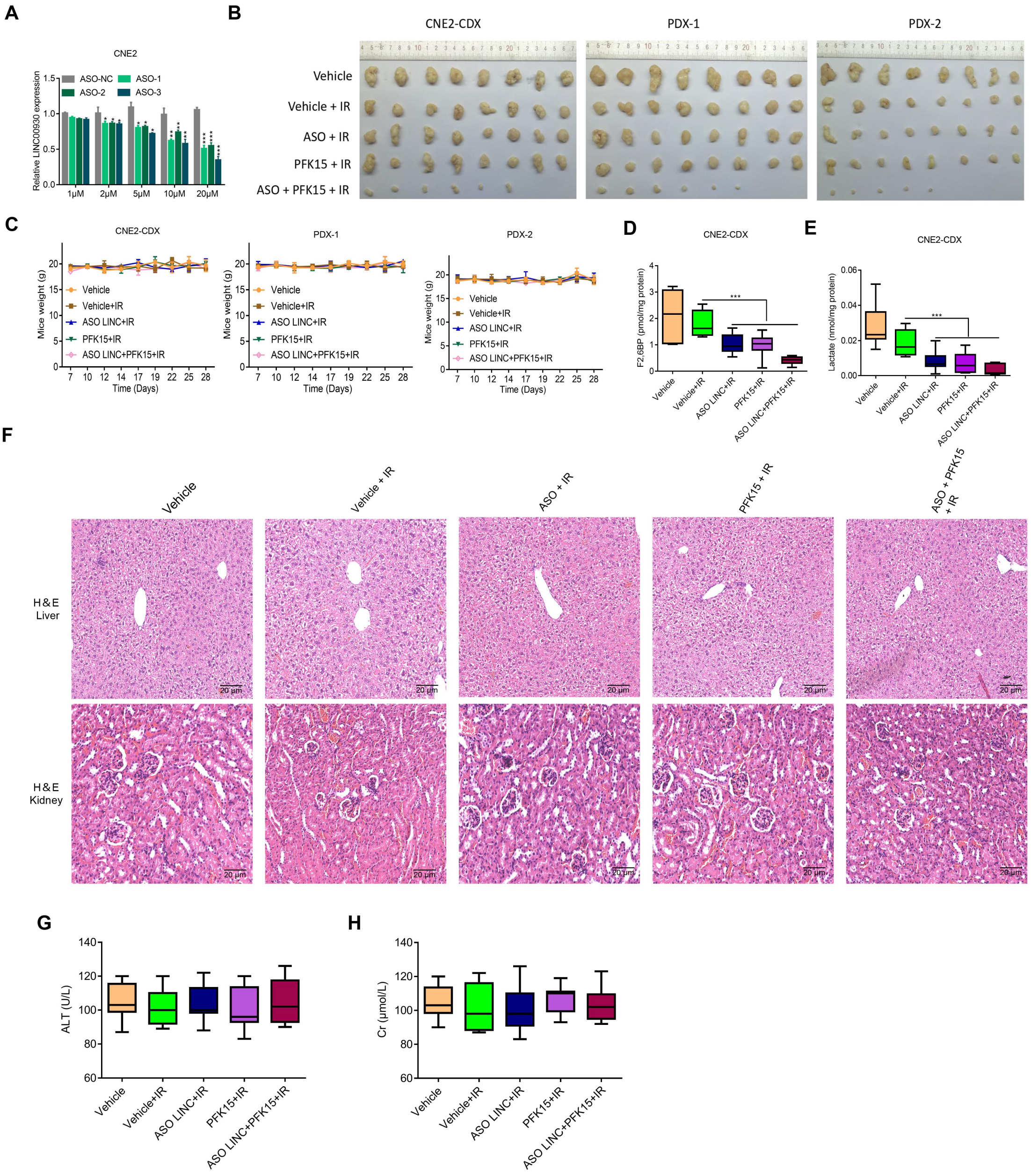

Figure S6 related to Fig. 6 and Fig. 7 Potential therapeutic role of LINC00930 in NPC. **a** QRT-PCR showed LINC00930 expression after ASO delivery without transfecting reagents. ASO were added into CNE2 cells at the concentration as indicated. After 48 h, RNA was extracted, and qRT-PCR was performed. **b** Images of xenograft tumors in therapeutic experiments. **c** Body weights of mice treated were maintained during the course of treatment. **d** and **e** F-2,6-BP and lactate levels were assayed in CNE2-CDX model. Three different sites were selected from each xenograft tumor (n = 8). **f** Representative IHC images of randomly selected human-derived tumors (PDX-1) from each group (100 ×). **g** Safety evaluation of

LINC ASO and PFK15 *in vivo*. H&E histology images of liver and kidney from tumor-bearing mice (PDX-1) after different treatments for four weeks (100×). **g** and **h** Serum levels of ALT and Cr in mice were assayed in therapeutic experiments. The p-value in **a, d & e** was determined by a two-tailed unpaired Student's *t* test. * $p < 0.05$; ** $p < 0.01$; *** $p < 0.001$.
